# Interactome analysis identifies a new paralogue of XRCC4 in non-homologous end joining DNA repair pathway

**DOI:** 10.1038/ncomms7233

**Published:** 2015-02-11

**Authors:** Mengtan Xing, Mingrui Yang, Wei Huo, Feng Feng, Leizhen Wei, Wenxia Jiang, Shaokai Ning, Zhenxin Yan, Wen Li, Qingsong Wang, Mei Hou, Chunxia Dong, Rong Guo, Ge Gao, Jianguo Ji, Shan Zha, Li Lan, Huanhuan Liang, Dongyi Xu

**Affiliations:** 1 State Key Laboratory of Protein and Plant Gene Research, School of Life Sciences, Peking University, 5 Yiheyuan Road, Beijing 100871, China; 2 State Key Laboratory of Biomacromolecules, Institute of Biophysics, Chinese Academy of Sciences, Beijing 100101, China; 3 State Key Laboratory of Virology, School of Basic Medicine, Wuhan University, Wuhan 430071, China; 4 Department of Microbiology and Molecular Genetics, University of Pittsburgh Cancer Institute, University of Pittsburgh School of Medicine, 5117 Centre Avenue, Pittsburgh, Pennsylvania 15213, USA; 5 Institute for Cancer Genetics, Columbia University Medical Center, New York City, New York 10032, USA

## Abstract

Non-homologous end joining (NHEJ) is a major pathway to repair DNA double-strand breaks (DSBs), which can display different types of broken ends. However, it is unclear how NHEJ factors organize to repair diverse types of DNA breaks. Here, through systematic analysis of the human NHEJ factor interactome, we identify PAXX as a direct interactor of Ku. The crystal structure of PAXX is similar to those of XRCC4 and XLF. Importantly, PAXX-deficient cells are sensitive to DSB-causing agents. Moreover, epistasis analysis demonstrates that PAXX functions together with XLF in response to ionizing radiation-induced complex DSBs, whereas they function redundantly in response to Topo2 inhibitor-induced simple DSBs. Consistently, PAXX and XLF coordinately promote the ligation of complex but not simple DNA ends *in vitro*. Altogether, our data identify PAXX as a new NHEJ factor and provide insight regarding the organization of NHEJ factors responding to diverse types of DSB ends.

## Abstract (web summary)

DNA double-strand breaks (DSBs), a highly deleterious form of DNA damage, are associated with multiple types of broken ends. Here, the authors identify a XRCC4-like factor that functions in the non-homologous end-joining DNA repair pathway to repair DSBs with complex broken ends.

## Graphical abstract

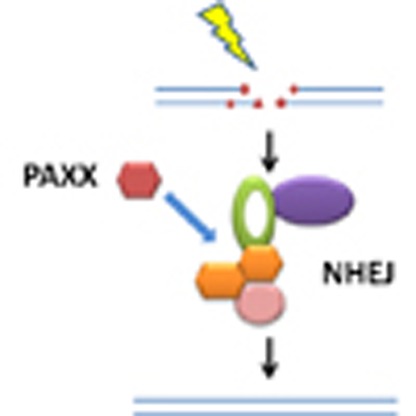

DNA double-strand breaks (DSBs), one of the most dangerous forms of DNA damage, are caused by aberrant replication and repair, programmed recombination and exposure to exogenous agents used in cancer therapies. Unrepaired DSBs can lead to apoptosis or senescence, whereas misrepaired DSBs can cause developmental defects, accelerated ageing and cancer^1^. Non-homologous end joining (NHEJ) is a major pathway for detection and repair of DSBs in mammalian cells^2^. The core factors mediating NHEJ are the Ku70/80 heterodimer (Ku), DNA-dependent protein kinase catalytic subunit (DNA-PKcs), XRCC4, DNA ligase IV (Lig4) and XRCC4-like factor (XLF, also called Cernunnos)^2^. For simple DSBs, these core factors are sufficient to recognize, align and ligate a pair of broken ends. However, for complex or ‘dirty’ breaks, which have mismatched or covalently modified DNA ends, additional factors are required to modify the ends and facilitate their ligation. These factors include Artemis, DNA polymerases λ and μ, terminal dinucletidyltrasferase, polynucleotide kinase-phosphatase, aprataxin (APTX) and APTX–polynucleotide kinase-phosphatase-like factor^1^. It remains unclear how these auxiliary factors are targeted to specific DSBs, in particular those with ends that contain densely clustered damage. It has been speculated that a fraction of NHEJ proceeds by a ‘trial and error’ process, in which sequential attempts at ligation and end processing by different enzymes are repeated until the substrate is cleaned for ligation^1^. This process requires a stable NHEJ complex to maintain the end-alignment. Many DSB repair proteins have been implicated in this role, including XRCC4 and its paralogue, XLF^3^.

The XRCC4-like proteins, including XRCC4, XLF and SAS6, function as homodimers composed of an amino-terminal globular head domain and a carboxy-terminal coiled-coil stalk. All of these proteins play important scaffold roles by forming various high-order structures^4^,^5^,^6^,^7^,^8^,^9^. XRCC4 and XLF can form super-helical filaments^6^,^7^,^8^,^9^, which have been shown to bridge, align or protect DNA ends^3^. In addition, they interact with Ku^10^ and Lig4 (refs 11, 12), respectively, and can stimulate Lig4 activities^13^,^14^,^15^. Moreover, XLF preferentially stimulates the end joining of non-cohesive ends over cohesive ends^16^,^17^, suggesting that it may be specifically needed for the repair of complex DNA ends.

We have previously identified Rif1 as a novel NHEJ regulator^18^. Here we perform systematic interactome analyses of human NHEJ factors and identify a novel XRCC4-like protein named paralogue of XRCC4 and XLF (PAXX). The crystal structure study reveals that PAXX forms a homodimer through its N-terminal domain that is structurally similar to XRCC4 and XLF. In contrast to XRCC4 and XLF, PAXX directly and strongly interacts with the Ku complex. Interestingly, PAXX and XLF cooperate to promote the ligation of non-cohesive but not blunt ends *in vitro*. Importantly, the epistasis analysis suggests that PAXX is upstream of XRCC4 and Lig4 in DSB repair, while PAXX and XLF display both epistatic and non-epistatic interactions that are dependent on the complexity of the DSB ends. Thus, our data reveal that PAXX is a new core NHEJ factor that plays a key role in the organization of NHEJ complexes for the repair of diverse types of DSB ends.

## Results

### PAXX is a new component of NHEJ

**Figure 1:**
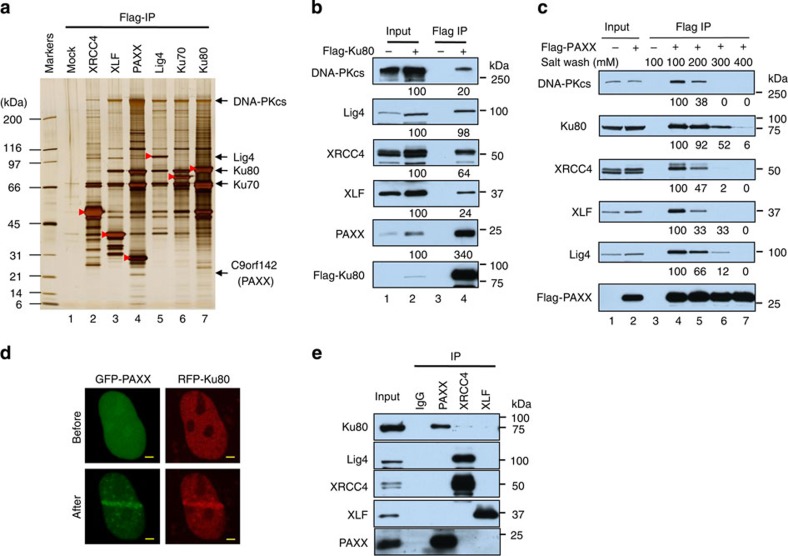
PAXX is one component of the NHEJ complex. (**a**) A silver-stained SDS gel showing the polypeptides that were immunopurified with Flag antibody from the extracts of HEK293 cells expressing Flag-tagged XRCC4, XLF, PAXX, Lig4, Ku70 or Ku80. As a control, a mock IP (Mock) was performed using regular HEK293 cells that did not express Flag-tagged protein. The proteins identified using mass spectrometry and the number of peptides discovered for each protein are listed in Table 1. The Flag-tagged bait proteins in the gel are indicated by red arrowheads. (**b**) Immunoblotting shows that PAXX co-immunoprecipitates with Flag-Ku80. (**c**) Immunoblotting shows Flag-PAXX IP under different wash conditions with the indicated sodium concentrations. The numbers in **b** and **c** indicate the related intensity of the bands above. (**d**) The accumulation of green fluorescent protein (GFP)-PAXX at the laser microirradiation-induced damage sites in U2OS cells. RFP-Ku80 was included as a control. Images were obtained before (left panel) and 3 min after (right panel) irradiation. Scale bars, 2 μm. (**e**) Immunoblotting of the IP with endogenous antibodies.

We systematically expressed 19 Flag-tagged NHEJ proteins in HEK293 cells and immunoprecipitated their associated complexes using an anti-Flag antibody (Fig. 1a, Table 1 and Supplementary Data 1). A silver-stained gel of partial samples confirmed the high quality of the immunoprecipitations (Fig. 1a). Mass spectrometry identified a novel protein, C9orf142, in the immunoprecipitates from 4 (XLF, Lig4, Ku70 and Ku80) of the 5 NHEJ core proteins (Fig. 1a, lanes 2, 3 and 5–7) and 2 (DNA polymerases λ and APTX–polynucleotide kinase-phosphatase-like factor) of the 14 non-core NHEJ proteins (Supplementary Data 1). Immunoblotting of the Ku80-associated complex further validated this finding (Fig. 1b). Based on the sequence and structural similarity to XRCC4 and XLF (see below), we renamed C9orf142 as PAXX. Silver staining (Fig. 1a, lane 4), mass spectrometry (Table 1) and immunoblotting (Fig. 1c) of the reciprocal immunoprecipitation (IP) revealed the presence of major NHEJ proteins, including XLF, XRCC4, Lig4, Ku70, Ku80 and DNA-PKcs in the PAXX-associated complexes, demonstrating that PAXX is a new component of the NHEJ complex.

NHEJ factors have been reported to relocalize to laser-induced DSB sites *in vivo*^19^. We expressed green fluorescent protein-fused PAXX in U2OS cells and induced DSBs with a micro-laser. Green fluorescent protein-PAXX relocalized to the DSB sites as expected (Fig. 1d), consistent with the biochemical results that PAXX is an NHEJ factor.

### PAXX forms a stable complex with Ku

We noted that PAXX is enriched between 3- and 17-fold over other core NHEJ proteins examined in the Ku80-associated complexes (Fig. 1b), suggesting that PAXX may interact with Ku more strongly than other NHEJ factors. In agreement with this notion, we found that the interaction between PAXX and Ku80 was more resistant to high salt treatment than those between PAXX and other NHEJ proteins (Fig. 1c, lanes 4–7). Moreover, immunoblotting of the immunoprecipitates of endogenous proteins showed that PAXX but not XRCC4 and XLF co-immunoprecipitated with Ku80 (Fig. 1e). Interestingly, the three members of the XRCC4 family did not co-immunoprecipitate with one another in detectable quantities under our experimental conditions, which is inconsistent with a previous report showing that XRCC4 and XLF co-immunoprecipitate^20^. These data suggest that PAXX forms a more stable complex with Ku than with XRCC4, XLF and other NHEJ core factors.

### PAXX is structurally similar to XRCC4 and XLF

**Figure 2:**
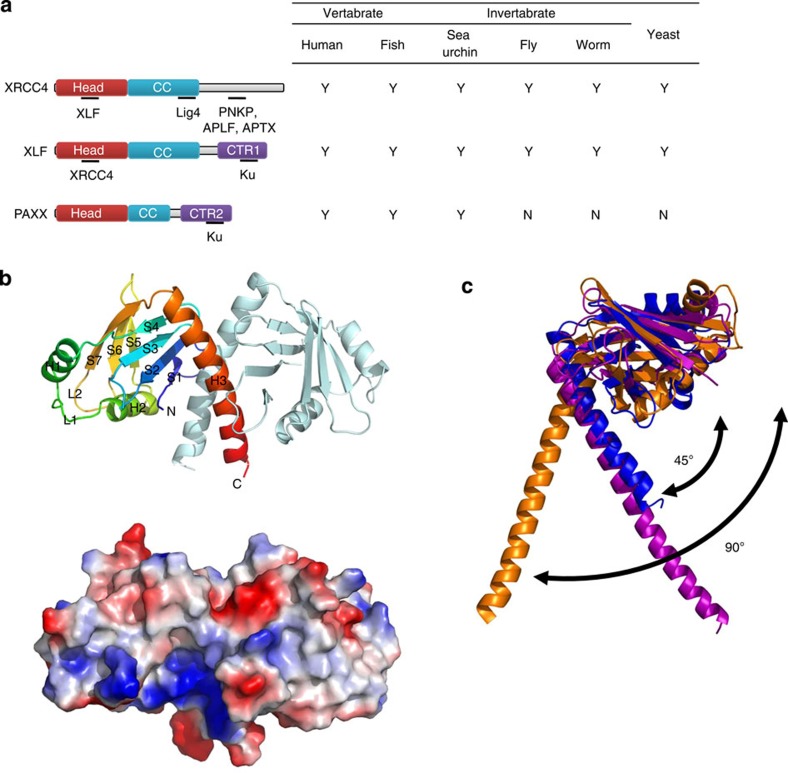
The N-terminal domain of PAXX is structurally similar to those of XRCC4 and XLF. (**a**) Domain maps of XRCC4, XLF and PAXX. The sites of protein–protein interactions are marked by underline and the interacting proteins are indicated below. Conversation of the three proteins is displayed in the right panel. The orthologues in human (*Homo sapiens*), fish (*Danio rerio*), sea urchin (*Strongylocentrotus purpuratus*), fly (*Drosophila melanogaster*), worm (*Caenorhabditis elegans*) and yeast (*Saccharomyces cerevisiae*) were identified using the BLASTP algorithm, to search the NR database maintained at NCBI. Y, exists; N, does not exist. Sequence alignments are shown in Supplementary Fig. 1. (**b**) The homodimer structure of PAXX AA 1–145. Top panel: the homodimer structure is shown in cartoon representation. One monomer is shown in spectrum and the other is shown in pale cyan. The spectrum ranging from blue to red represents residues from the N to C terminus. The N terminus, C terminus and secondary structural elements are labelled. Bottom panel: electrostatic potential surface representation of PAXX dimer. The positive, negative and neutral regions are shown in blue, red and white, respectively. (**c**) Overlay of N-terminal head domains of PAXX AA 1–145, XLF AA 1–169 (PDB 2QM4) and XRCC AA 41–164 (PDB 1IK9), in blue, orange and purple, respectively.

Sequence homology searches using the BLAST algorithm showed that PAXX has no obvious similarity to any known proteins in the NCBI database. However, three-dimensional structure prediction with HHprep (http://toolkit.tuebingen.mpg.de/hhpred#) suggested that PAXX belongs to the same family as XRCC4 and XLF (Fig. 2a and Supplementary Fig. 1a,b). Specifically, PAXX protein is predicted to possess an N-terminal domain structure that is identical to XRCC4, XLF and SAS6 (Fig. 2a and Supplementary Fig. 1a,b); all of these proteins contain a globular head domain followed by a coiled-coil stalk^4^,^5^,^21^,^22^. To test this hypothesis, we determined the crystal structure of the PAXX N-terminal fragment (1–145) diffracted at 2.6 Å (PDB 4WJA) using the SAD (single wavelength anomalous scattering) and MR (molecular replacement) methods (Fig. 2b and Table 2). The space groups for both data sets were P6_5_22, with two molecules in each asymmetric unit. As predicted, the N-terminal domain of PAXX was highly similar to those of XLF and XRCC4 (Fig. 2b and Supplementary Fig. 1b). Seven β-strands (S1–S7) and three helixes (H1–H3) were observed in the PAXX 1–145 structure, in which a globular head domain and a coiled-coil stalk formed a dimer (Fig. 2b), consistent with the observation that PAXX 1–145 exists primarily as a dimer in solution (Supplementary Fig. 2).

Compared with XLF, PAXX showed more structural similarities to XRCC4 (Fig. 2b,c). Different values for the angle between the head domain and H3 have been reported for XLF and XRCC4. The C terminus of XLF folds back to the linkage point between the head domain and H3, and elevates the head domain, forming a 90° angle between them (Fig. 2b)^21^. In XRCC4 and PAXX, the angle between the head domain and H3 is only ~45° due to a lack of tension (Fig. 2c)^22^. In addition, compared with XRCC4 and XLF, PAXX has a much shorter coiled-coil stalk (Fig. 2c and Supplementary Fig. 1b). The extended stalk of XRCC4 is required for Lig4 binding, implicating that the N-terminal domain of PAXX may lack the ability to bind Lig4.

The database of the Human Protein Atlas (http://www.proteinatlas.org/) shows that PAXX is widely expressed in all human tissues, with particularly high expression in the blood and immune system. PAXX is present in all vertebrates but is absent in most invertebrates and in yeast (Fig. 2a). This evolutionary distribution of PAXX differs from that of XRCC4 and XLF, which are conserved from yeast to vertebrates. PAXX appears to co-evolve with DNA-PKcs and Artemis, which implies that PAXX may function in a manner similar to DNA-PKcs and Artemis in joining the complex but not the simple DNA ends^23^.

### PAXX interacts with NHEJ factors via its C-terminal region

**Figure 3:**
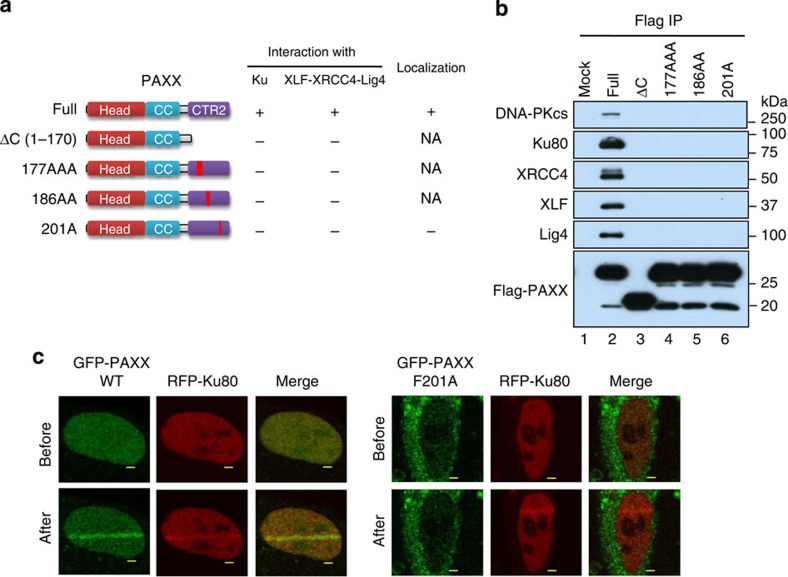
PAXX interacts with the Ku complex and the XLF–XRCC4–Lig4 complex through its CTR. (**a**) Schematic representation of different PAXX mutants (left) and their ability to coimmunoprecipitate with Ku and XLF–XRCC4–Lig4 complexes from HEK293 extracts (right). Mutation sites are indicated in Supplementary Fig. 1a. (**b**) An IP–WB analysis to determine whether PAXX mutants in **a** coimmunoprecipitate with different NHEJ proteins. (**c**) Imaging shows that the PAXX mutant F201A loses its localization at laser microirradiation-induced damage sites. RFP-Ku80 was included as a control. Images were obtained before and 3 min after irradiation. Scale bars, 2 μm.

Unlike the N-terminal domain structure that is shared among XRCC4 family members, C-terminal regions (CTRs) are more divergent and show little sequence similarity (Fig. 2a). The C-terminal-conserved region of PAXX, similar to that of XLF, localizes at the end of C terminus (Fig. 2a and Supplementary Fig. 1a)^10^. A previous study has shown that the CTR domain of XLF is required for XLF to interact with Ku and XRCC4, and for the localization at DSBs^10^. We found that the CTR motif of PAXX is similarly required for these associations, because the deletion (AA 1–170) and point mutation (RRR177–179AAA, IN186–187AA or F201A) of the PAXX-CTR completely abolished its interaction with Ku and XRCC4, as well as Lig4 and DNA-PKcs (Fig. 3a,b and Supplementary Fig. 1a). Moreover, the F201A mutation also disrupted the localization of PAXX at laser-induced DSBs and compromised its exclusively nuclear localization (Fig. 3c). These data support the notion that PAXX and XLF may interact with common factors in the NHEJ pathway and imply that the two proteins may have overlapping functions.

### PAXX promotes cellular resistance to agents that induce DSBs

**Figure 4:**
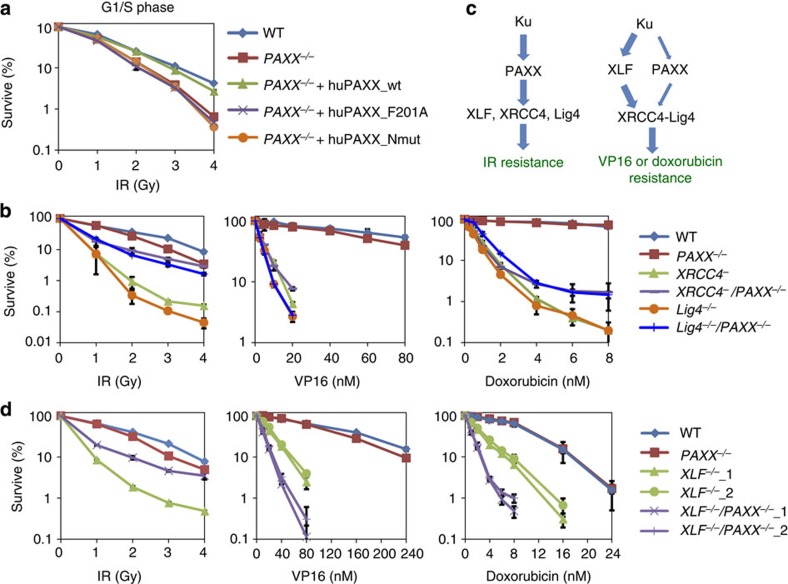
PAXX works in both the same and parallel DNA repair pathways with XLF. (**a**) Sensitivity curves for *PAXX*^*−/−*^ DT40 cells complemented by wild-type, F201A or Nmut variants of human PAXX in G1/S phase. Cells were arrested in G1/S phase before IR treatment. Mutant sites in Nmut are indicated in Supplementary Fig. 1a. (Sensitivity assays to analyse genetic interactions of PAXX with XRCC4, Lig4 (**b**) and XLF (**d**) in asynchronous DT40 cells. Survival curves were measured using MTS assay. The mean and s.d. from three independent experiments are shown. (**c**) A cartoon showing potential genetic relationships of PAXX with XRCC4, Lig4 and XLF under different treatment conditions.

To investigate the function of PAXX *in vivo*, we generated *PAXX*-null DT40 cells by gene targeting (Supplementary Fig. 3a,b). These cells displayed a modest sensitivity to a variety of agents that induce DSBs, including ionizing radiation (IR), bleomycin (a radiomimetic agent that can induce a greater number of homogeneous DSBs than IR^24^), VP16 (etoposide, a Topoisomerase II (Topo2) inhibitor that directly induces DSBs with less complex ends^25^,^26^) and ICRF193 (a Topo2 inhibitor that indirectly induces DSB via an unknown mechanism; Supplementary Fig. 4), demonstrating that PAXX is required for normal DSB repair *in vivo*. As NHEJ-deficient cells are much more sensitive to IR when arrested in G1 or early S phase^27^, we analysed *PAXX*^*−/−*^ cells under these conditions and found that they are hypersensitive to IR (Fig. 4a), consistent with the idea that PAXX plays a role in NHEJ. Among other DSB-inducing agents, ICRF193 sensitivity is usually observed in cells defective in NHEJ but not homologous recombination (HR) pathways^28^. The finding that *PAXX*^*−/−*^ cells possess ICRF193 sensitivity (Supplementary Fig. 4) is consistent with the biochemistry data, suggesting that PAXX plays a role in NHEJ. Conversely, *PAXX*^*−/−*^ cells showed no obvious sensitivity to camptothecin (Supplementary Fig. 4), a Topoisomerase I inhibitor that induces replication-dependent DSBs that are repaired primarily by the HR pathway^29^. These data are consistent with the notion that PAXX participates in NHEJ but not HR. We further examined the function of PAXX in the DSB repair in mammalian cells and generated PAXX-deficient human HCT116 cells using CRISPR (Supplementary Fig. 5a,b). Consistent with the results in DT40 cells, PAXX-deficient HCT116 cells were hypersensitive to IR and VP16 (Supplementary Fig. 5c), which suggested that PAXX is also important for the DSB repair pathways in mammalian cells.

### Both N- and C-terminal domains are required for PAXX function

To determine which domain is important for the function of PAXX in DSB repair, we performed genetic rescue experiments using *PAXX*^*−/−*^ DT40 cells transfected with variants of PAXX. Re-expression of wild-type human PAXX protein in *PAXX*^*−/−*^ cells largely rescued the IR sensitivity phenotype, whereas re-introduction of the Ku-interaction-deficient mutant PAXX (F201A) did not, which indicated that Ku-binding activity is critical for the function of PAXX in DSB repair (Fig. 4a).

The S6-loop-S7 region of the global head domain in XRCC4 or XLF is important for their mutual interactions and for their functions in NHEJ^6^,^7^,^8^,^9^. We generated a combined mutation (Nmut: L96D, L98D, L105D and L109D) in this region of PAXX (Supplementary Fig. 1a), which is expected to disrupt the hydrophobic interface. Nmut also failed to rescue the IR sensitivity phenotype (Fig. 4a), suggesting that both the N- and C-terminal domains are important for PAXX promotion of DSB repair.

### PAXX acts upstream of the XRCC4–Lig4 complex

To examine how PAXX interacts genetically with other NHEJ factors, we performed an epistasis analysis by generating *Lig4*^*−/−*^, *XRCC4*^*−*^ and *XLF*^*−/−*^ single-mutant DT40 cells and then inactivating PAXX in these cells to create double knockouts (Supplementary Fig. 3c–h). In agreement with an earlier report, XRCC4 and Lig4 single knockout cells displayed a strong hypersensitivity to IR (Fig. 4b, left panel)^30^. The IR sensitivity was not as severe in *PAXX*^*−/−*^ cells as in *XRCC4*^*−*^ and *Lig4*^*−/−*^ cells, suggesting that PAXX is not as essential as XRCC4 and Lig4 for DSB repair. Interestingly, the inactivation of PAXX dramatically suppressed IR sensitivity in both *Lig4*^*−/−*^ and *XRCC4*^*−*^ cells (Fig. 4b, left panel) and also partially suppressed the slow proliferation phenotype of *Lig4*^*−/−*^ cells (Supplementary Table 1). This phenotype mimics that of Ku70, the mutation of which also suppresses the IR sensitivity of *Lig4*^*−/−*^ cells^30^. As Ku functions upstream in the NHEJ pathway, these data imply that PAXX may, similar to Ku, function upstream of the XRCC4–Lig4 complex (Fig. 4c, left panel). These data are also consistent with the results of our biochemistry analysis, which indicated that PAXX has a more stable interaction with Ku than with other NHEJ core factors.

Following treatment with the DSB-inducing agents bleomycin, ICRF193 and VP16, *Lig4*^*−/−*^ and *XRCC4*^*−*^ single-mutant cells did not differ significantly in terms of sensitivity compared with their respective PAXX double mutant cells (Fig. 4b, middle panel and Supplementary Fig. 6a,b), which suggested that PAXX functions in the same NHEJ pathway as XRCC4 and Lig4 to repair DSBs caused by these reagents (Fig. 4c, right panel).

### PAXX and XLF function in both common and parallel pathways

The *PAXX*^*−/−*^*/XLF*^*−/−*^ double-mutant cells, such as the *PAXX*^*−/−*^*/XRCC4*^*−*^ and *PAXX*^*−/−*^*/Lig4*^*−/−*^ cells, showed reduced IR sensitivity compared with the *XLF*^*−/−*^ single-mutant cells, indicating that PAXX also functions upstream of XLF in the repair of IR-induced DSBs (Fig. 4c,d, left panels). Similarly, *XLF*^*−/−*^ single- and *PAXX*^*−/−*^*/XLF*^*−/−*^ double-mutant cells showed no significant differences in bleomycin and ICRF193 sensitivity (Supplementary Fig. 6c), suggesting that PAXX and XLF function in the same pathway to protect against cell death caused by these agents. Importantly, the *PAXX*^*−/−*^*/XLF*^*−/−*^ double-mutant cells displayed an increased sensitivity to VP16 than did either one of the single-mutant cells (Fig. 4d, middle panel). To test whether this phenomena was due to the different cell survival assays for IR and VP16, we also examined the VP16 sensitivity using a colony formation assay. The results were similar to those obtained using the MTS (3-(4,5-dimethylthiazol-2-yl)-5-(3-carboxymethoxyphenyl)-2-(4-sulfophenyl)-2H-tetrazolium) assay (Supplementary Fig. 7). Moreover, the double-mutant cells also displayed a slower proliferation than each single mutant (Supplementary Table 1). Together, these data suggest that the two XLF paralogues act in parallel pathways to resist VP16-induced cell death and to promote cell proliferation.

To study whether PAXX and XLF work in parallel pathways to resist other DSB-causing agents, we assayed the effects of doxorubicin, a Topo2 inhibitor similar to VP16. We found that the *PAXX*^*−/−*^*/XLF*^*−/−*^ double-mutant cells displayed increased sensitivity to doxorubicin than either of the single-mutant cells (Fig. 4d, right panel). By comparison, the doxorubicin sensitivity of *PAXX*^*−/−*^*/XRCC4*^*−*^ and *PAXX*^*−/−*^*/Lig4*^*−/−*^ double-mutant cells was comparable to that of the *XRCC4*^*−*^ or *Lig4*^*−/−*^ single-mutant cells (Fig. 4b, right panel). These data suggest that PAXX acts in a parallel pathway to XLF but in the same pathway as XRCC4 and Lig4, to repair DSBs caused by Topo2 inhibitors (Fig. 4c, right panel).

### PAXX forms a complex with DNA-bound Ku

**Figure 5:**
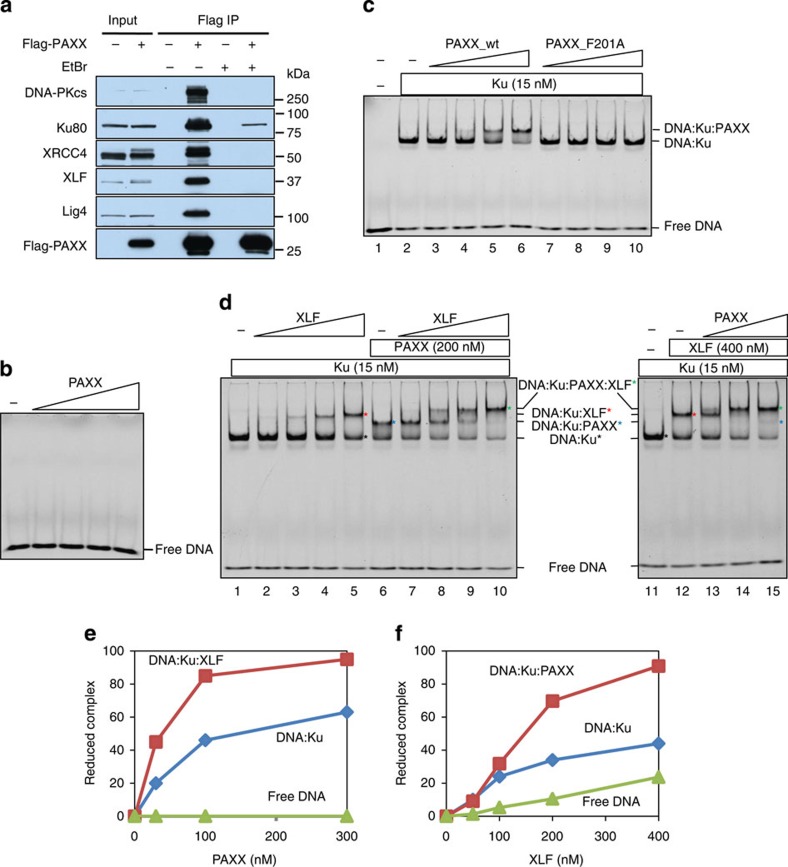
PAXX promotes the assembly of XLF into the DNA end-bound Ku complex. (**a**) Immunoblotting showing Flag-PAXX with or without EtBr. (**b**) Electrophoretic mobility shift assay (EMSA) showing that PAXX does not bind DNA. Reactions contain 5 nM Cy3-labelled 30-bp dsDNA and 10, 30, 100 or 300 nM PAXX. (**c**) EMSA assay showing that PAXX but not its mutant F201A supershifts the DNA–Ku complex. Reactions were performed as in **b**, excluding the presence of Ku in the indicated wells. The composition of the protein–DNA complexes is indicated on the right side. (**d**) EMSA showing that PAXX and XLF mutually promote their recruitment to the DNA–Ku complex. Reactions contain 5 nM Cy3-labelled 30-bp dsDNA and the indicated proteins. The series of concentrations was 50, 100, 200 and 400 nM for XLF, and 30, 100 and 300 nM for PAXX. (**e**,**f**) Quantifications of reduced complexes. The reduced complexes in **d**, lanes 6–10 (**f**) and lanes 11–15 (**e**) were quantified. The complex quantities in lanes 6 and 11 were set at 100% for the respective experiments.

It has been shown that the interaction between XLF and the Ku complex is mediated by DNA^31^. We found that the addition of ethidium bromide, which intercalates into DNA and interferes with DNA–protein interactions, to cell lysates strongly reduced the association between PAXX and other NHEJ proteins, including Ku (Fig. 5a). These data suggest that PAXX and XLF share another common feature—they both use DNA to mediate their interactions with Ku.

We investigated how DNA mediates the interaction between PAXX and Ku. One possibility is that DNA acts as a bridge between proteins. We do not favour this possibility, because PAXX showed no detectable binding activity for DNA up to 315 bps in length (Fig. 5b and Supplementary Fig. 8a,b). This feature differs from its paralogues XRCC4 and XLF, which possess length-dependent DNA-binding activity^14^,^15^ (see DNA-binding activity of XLF in Supplementary Fig. 8c,d). The finding that PAXX differs from its paralogues in terms of the DNA-binding characteristics implies that it may have a unique function in the NHEJ pathway.

Another mechanism by which DNA may facilitate the PAXX–Ku interaction is that DNA binding may alter the conformation of Ku to favour an interaction with PAXX. One prediction of this hypothesis is that PAXX would be capable of interacting with the DNA end-bound Ku complex. Consistent with this prediction, PAXX supershifted the DNA-bound Ku complex (Fig. 5c lane 2) but not free DNA in electrophoretic mobility shift assays (Fig. 5c, lanes 3–6). As a control, no supershift was observed for the PAXX-CTR domain mutant F201A, which is defective in its association with Ku in co-IP assays (Fig. 5c, lanes 7–10). These results suggest that PAXX can interact with DNA-bound Ku through its CTR domain to form a DNA–Ku–PAXX tertiary complex at DNA ends. This feature resembles that of XLF, which can form similar DNA–Ku–XLF tertiary complexes^31^. Thus, the two XLF paralogues form similar complexes at DNA ends to promote DNA end joining, which may explain why they have redundant functions in repairing Topo2 inhibitor-induced DSBs.

### PAXX promotes the assembly of the NHEJ complex

The above data suggest that XLF and PAXX can each form a tertiary complex with DNA-bound Ku. This phenomenon prompted us to investigate whether each protein can be assembled into the tertiary complex pre-formed by its paralogue to produce a quaternary complex. Consistent with a previous report 31, XLF supershifted the band corresponding to the DNA-bound Ku complex to produce a band corresponding to the DNA–Ku–XLF tertiary complex (Fig. 5d, lanes 2–5 and lane 12). PAXX further supershifted this band to produce a slower mobility band, which probably represents the DNA–Ku–XLF–PAXX quaternary complex (Fig. 5d, lanes 13–15 and Fig. 5e). Notably, the addition of PAXX significantly reduced the amount of tertiary complex but not of the DNA–Ku complex (Fig. 5e), suggesting that the incorporation of XLF into the DNA–Ku complex promotes the subsequent binding of PAXX. In the reciprocal experiment, XLF similarly supershifted a preformed DNA–Ku–PAXX tertiary complex (Fig. 5d, lane 6) to produce the DNA–Ku–XLF–PAXX quaternary complex (Fig. 5d, lanes 7–10). Moreover, XLF substantially reduced the amount of tertiary complex but only modestly affected the amount of the binary (DNA–Ku) complex (Fig. 5d, lanes 7–10 and Fig. 5f), indicating that the assembly of PAXX into the DNA–Ku complex also facilitated subsequent binding by XLF. Together, these data suggest that the two XLF paralogues can cooperate to assemble a DNA–Ku–XLF–PAXX quaternary complex at DNA ends. This quaternary complex is more stable than either tertiary complex, which explains why both XLF and PAXX are required for a stable NHEJ complex to repair IR-induced DSB ends.

### PAXX stimulates the ligation of non-cohesive DNA ends

**Figure 6:**
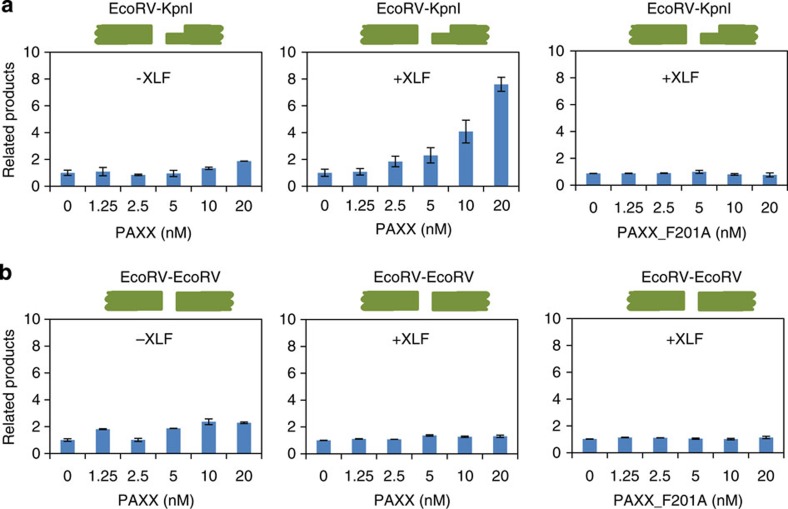
PAXX promotes non-cohesive end joining. (**a**,**b**) PAXX stimulates non-cohesive end ligation in the presence of XLF. The linear DNA substrates contain blunt–3′ overhangs (EcoRV–KpnI) (**a**) or blunt–blunt (EcoRV–EcoRV) (**b**) ends. The reactions contain 5 nM Ku70/80, 2.5 nM XRCC4–Lig4 and the indicated wild-type PAXX or its mutant F201A, with or without 2.5 nM XLF. Data from three independent experiments are represented as the mean±s.e.m.

XLF has been shown to robustly stimulate the ligation of mismatched and non-cohesive DNA ends *in vitro*^16^. We found that the presence of PAXX had no significant effect on the ligation of DNA substrates with non-cohesive ends (EcoRV–KpnI overhangs) using the same assay (Fig. 6a, left panel). As a control, XLF strongly enhanced this ligation reaction (11.4-fold at 2.5 nM concentration; Supplementary Fig. 9). Interestingly, in the presence of 2.5 nM XLF, PAXX stimulated the ligation by 7.6-fold (Fig. 6a, middle panel), demonstrating that PAXX has an XLF-dependent stimulatory effect on the ligation of non-cohesive DNA ends. Moreover, this stimulatory activity was not detected for the PAXX-F201A mutant (Fig. 6a, right panel), which has a deficient association with Ku. This characteristic resembles that of XLF^32^, which suggests that the interaction with Ku is essential for both XLF paralogues to stimulate the ligation of DNA ends. Furthermore, neither PAXX nor its mutant (F201A) had a significant effect on the ligation of blunt DNA ends (EcoRV–EcoRV) in the absence or the presence of XLF (Fig. 6b), which implies that PAXX preferentially stimulates the joining of complex but not simple DNA ends.

## Discussion

In this study, we performed interactome analyses of the human NHEJ pathway and identified PAXX as a new component of this pathway. PAXX stably interacts with the DNA end-bound Ku complex, cooperates with XLF to stimulate non-cohesive DNA end ligation *in vitro* and is required for cellular resistance to DSB-causing agents *in vivo*. Genetic analyses further demonstrated that PAXX and the XRCC4–Lig4 complex work in a common pathway to repair DSBs induced by IR, bleomycin and Topo2 inhibitors.

PAXX shares structural and functional similarities with its paralogues XRCC4 and XLF; they all possess the same N-terminal domains, can stably assemble into a DNA-end-bound NHEJ complex, can rapidly relocalize to DSBs and can promote cellular resistance to DSB-inducing agents. Structurally, the N-terminal domain of PAXX is more similar to that of XRCC4, as both exhibit a 45° angle between the head domain and the helix stalk. Conversely, the C-terminal domain of PAXX is more similar to that of XLF, as indicated by the fact that both can directly interact with DNA-bound Ku.

The finding that the inactivation of PAXX, XRCC4 and XLF in DT40 cells resulted in cellular hypersensitivity to agents causing DSBs indicates that each protein must have at least one unique role in the NHEJ pathway that cannot be substituted by its paralogues. One unique feature of PAXX is that it shows a strong association with Ku but no detectable direct association with Lig4. This characteristic differs from XRCC4, which stably associates with Lig4, and from XLF, which weakly associates with both Ku and XRCC4. Another unique feature of PAXX is that it is present in vertebrates but absent in yeast and most invertebrates, unlike XRCC4 and XLF, which are present in all eukaryotes. Another NHEJ factor that shows a similar evolutionary distribution is DNA-PKcs, which mediates the joining of complex but not simple ends in V(D)J recombination^23^. It is possible that PAXX co-evolved with DNA-PKcs to repair DSBs with more sophisticated ends in higher eukaryotes.

The observation that PAXX and Ku can form a stable complex suggests that they work together in the NHEJ pathway. Consistent with this suggestion, our genetic data showed that similar to Ku, PAXX may act upstream of the XRCC4–Lig4 complex. This hypothesis is supported by the finding that mutations in PAXX suppress the IR sensitivity of XRCC4 or Lig4 mutant DT40 cells (Fig. 4b), which mimics previous findings that the Ku mutation suppresses the phenotype of Lig4 mutations in DT40 cells and mice^30^,^33^. One model to explain the observed genetic suppression is that without Lig4 or XRCC4, the NHEJ pathway is inactive and DSBs are channelled to alternative DSB repair pathways, including HR and microhomology-mediated end joining^30^,^33^. However, binding of the PAXX–Ku complex to DNA ends protects the ends against resection, and thus inhibits the alternative pathways. The deletion of either PAXX or Ku releases the block and allows resection and alternative repair reactions to occur, thus suppressing the mutant phenotypes of Lig4 or XRCC4.

**Figure 7:**
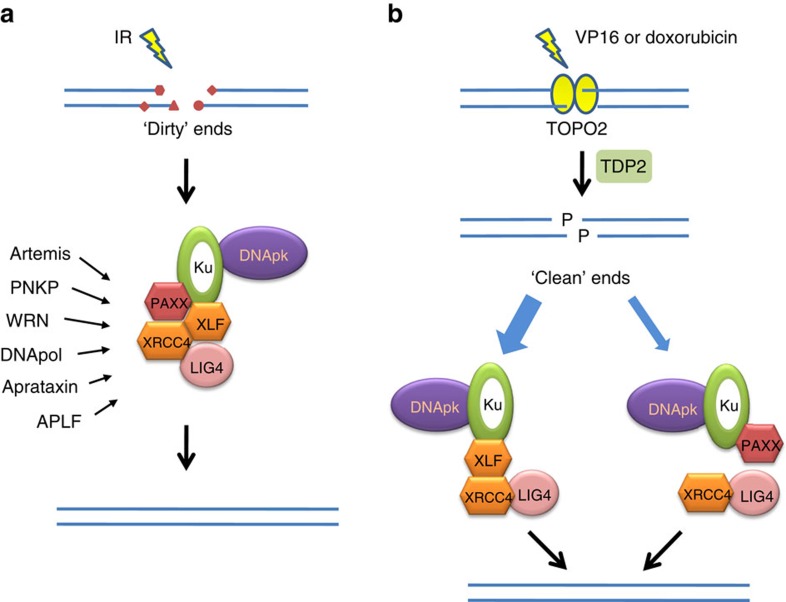
A model showing the relationship of PAXX and XLF in DSB repair. (**a**) IR induces DSBs with complex ends containing multiple modifications (‘dirty ends’). Cells require a full NHEJ complex, including both PAXX and XLF, to stabilize the alignment of ends for a ‘trial and error’ process with multiple modification enzymes. (**b**) A Topo2 inhibitor, VP16 or doxorubicin, will induce the formation of a Topo2–DNA cross-linked complex. The Topo2 peptide will be removed by TDP2 to generate a cohesive end that is phosphorylated at its 5′-end. These ‘clean’ ends can be quickly ligated even by an incomplete NHEJ complex lacking XLF or PAXX.

PAXX is closely related to XLF; both proteins can interact with the DNA end-bound Ku complex through their C-terminal domains and stimulate the ligation of non-cohesive DNA ends. Interestingly, genetic studies have revealed that the two proteins work through different mechanisms to promote cellular resistance to different types of DSBs induced by various agents. For IR-induced DSBs that carry DNA ends that are more complex or ‘dirty’ (with many chemical modifications)^24^, the two proteins work in the same pathway (Fig. 7a). However, for DSBs with DNA ends that are ‘clean’ or have fewer modifications, such as those produced by Topo2 inhibitors^25^,^26^ and processed by TDP2 (ref. 34; Fig. 7b), the two proteins act in parallel pathways. One hypothesis to explain the difference is that the ‘dirty’ ends require a more stable complex to maintain their alignment and to allow a ‘trial and error’ process by different enzymes to remove the complex covalent modifications^1^. Both PAXX and XLF may be required to constitute such a stable complex (Fig. 7a). PAXX may first be recruited to DNA-bound Ku, because PAXX has a more stable association with the Ku complex than XLF (Fig. 1b,c,e). This process results in the subsequent recruitment of XLF to form the PAXX–DNA–Ku–XLF complex, which stabilizes the complex and enables the assembly of other NHEJ factors. For ‘clean’ ends, the ‘trial and error’ process is not needed and, therefore, one XLF or PAXX protein may be sufficient to maintain the end alignment for ligation (Fig. 7b). This finding is consistent with our biochemical observation that PAXX and XLF can be assembled individually into a DNA-bound Ku complex (Fig. 5c,d). In support of this hypothesis, our biochemistry data showed that PAXX and XLF cooperated to stimulate the ligation of complex (non-cohesive) but not simple (blunt) DNA ends. Thus, PAXX and XLF have both cooperative and redundant functions that are dependent on the complexity of the DSB ends.

Altogether, our data provide new insight into the process of NHEJ factor organization and assembly in response to different DSBs that arise in a variety of ways.

## Methods

### Cell culture and transfection

HeLa, U2OS, HCT116 and HEK293 cells were purchased from ATCC. DT40 cells were a gift from the laboratory of Dr Shunichi Takeda. HeLa and U2OS cells were cultured in DMEM supplemented with 10% FCS at 37 °C with 5% CO_2_. HCT116 cells were cultured in RPMI1640. Suspension HEK293 cells were cultured in Freestyle medium (Invitrogen) plus 1% Gibco FCS and 1% glutamine in an incubator with shaking at 130 r.p.m. Plasmid transfections were conducted with Lipofectamine 2000 (Invitrogen) and PEI (polyethyleneimine, Polysciences) for U2OS and suspension HEK293 cells, respectively.

DT40 cells were cultured in RPMI 1640 medium supplemented with 10% FCS, 1% chicken serum, 10 mM HEPES and 1% penicillin–streptomycin mixture at 39.5 °C with 5% CO_2_. Transfection was performed by electroporation using the Lonza Nucleofector 4D. For selection, growth medium containing G418 (2 mg ml^−1^), puromycin (0.5 μg ml^−1^), Blasticidin (25 μg ml^−1^) or histidinol (1 mg ml^−1^) was used.

### Antibodies

Rabbit PAXX polyclonal antibodies were generated against an MBP-fused protein containing full-length PAXX and were used at dilution of 1:1,000 for western blotting (WB). Anti-Ku80 mouse monoclonal antibody was purchased from Santa Cruz (sc-56132, 1:200 for WB), anti-XLF from Bethyl (A300–730A, 1:1,000 for WB), anti-β-actin mouse monoclonal antibody from MBL (M177-3, 1:2,000 for WB), anti-XRCC4 rabbit polyclonal antibodies from Abcam (ab145, 1:1,000 for WB), anti-DNA-PKcs goat polyclonal antibodies from NeoMarkers Fremont (423 × 812, 1:1,000 for WB), anti-Lig4 rabbit polyclonal antibodies from Proteintech Group (12695-I-AP, 1:1,000 for WB) and anti-Flag M2 monoclonal antibody from Sigma-Aldrich (F3165, 1:1,000 for WB). Uncropped WBs are shown in Supplementary Fig. 10.

### Immunoprecipitation

The complementary DNAs of The NHEJ genes were obtained from hORFemone (V8.1). Mammalian expression plasmids were generated by shuttling cDNAs to the destination plasmid pDEST26-Flag using an LR reaction (Invitrogen).

For immunoprecipitation, suspension HEK293 cells were transfected with expression plasmids using PEI. After 64 h, the cells were harvested and the pellets were directly lysed with NTEN buffer (20 mM Tris-HCl, pH 7.5, 150 mM NaCl, 10% glycerol, 0.5% NP40, 10 mM NaF, 1 mM phenylmethylsulfonyl fluoride (PMSF), 1 μg ml^−1^ leupeptin, 1 μg ml^−1^ aprotinin). Following ultra-centrifugation at 440,000*g* for 15 min at 4 °C, the supernatant was incubated with anti-Flag M2-conjugated agarose beads for 3~4 h. The beads were then spun down and washed four times with IP buffer (20 mM Tris-HCl, pH 7.5, 150 mM NaCl, MgCl_2_ 5 mM, 10% glycerol, 0.1% NP40, 1 mM dithiothreitol (DTT) and 1 mM PMSF). Subsequently, the complexes were eluted with IP buffer containing 400 μg ml^−1^ 3 × Flag peptide and analysed by SDS–PAGE and mass spectrometry.

### Protein purification

For XLF, PAXX and its mutants, glutathione *S*-transferase-fused proteins were expressed in *Escherichia coli* (*Transetta* cells, TransGen) from a pCPD9 vector. Cells were grown at 37 °C until an OD600=0.8 and induced with 0.3 mM isopropylthiogalactoside at 28 °C for 3 h. The cell pellet from the 0.5-l culture was lysed using a French Press in 30 ml of PBST buffer (PBS buffer (10 mM phosphate, pH 7.4, 137 mM NaCl and 2.7 mM KCl) supplemented with 10% glycerol, 0.1% Triton100, 0.2 mM DTT and 0.5 mM PMSF). The lysate was centrifuged at 35,000*g* for 25 min and the supernatant was incubated with 1 ml Glutathione Sepharose 4B beads (GE Healthcare) at 4 °C for 3 h. The beads were washed twice with high-salt PBST buffer (500 mM NaCl) and twice with PBST buffer. Protein was eluted using TEV (50 μg ml^−1^) in TEV cleavage buffer (10 mM Tris Cl, pH 8.0, 100 mM NaCl, 0.5 mM EDTA, 0.01% NP40, 10% glycerol and 1 mM DTT) and loaded on a Superdex 75(10/300) column (GE Healthcare) that had been pre-equilibrated with buffer (50 mM Tris, pH 7.4, 0.5 M NaCl, 1 mM EDTA, 10% glycerol, 10 mM β-mercaptoethanol). Peak fractions containing the purified protein were flash-frozen and stored at −80 °C.

XRCC4–Lig4 and Ku70/80 complexes were purified as described^35^. Briefly, Sf9 insect cells infected with baculoviruses containing XRCC4 and Ligase IV-His (a gift from Dale Ramsden) were lysed, and XRCC4–Lig4 was purified with Ni-NTA beads (GE Healthcare), followed by Superdex-200 (26/60) and Mono Q (5/50 GL) columns (GE Healthcare). Ku was purified from the lysate of Sf9 cells expressing Ku70-His and Ku86 (a gift from Dale Ramsden) using Ni-NTA beads (GE Healthcare) and by gel filtration using a Superose-12 (HR 10/30) column (GE Healthcare).

For crystallization, PAXX 1–145 protein with a His_6_-tag at its N terminus was expressed using the pETDuet-1 vector in *E. coli*. The expression of the PAXX 1–145 protein was induced using 0.2 mM isopropylthiogalactoside at 16 °C. The pelleted cells were suspended in binding buffer (20 mM Tris-HCl, pH 8.0, 200 mM NaCl) and crushed by sonication. After centrifugation at 35,000*g* for 30 min at 4 °C, the supernatant was gently mixed with nickel beads (GE Healthcare). After elution from the nickel beads, PAXX 1–145 protein was further purified using a Q ion exchange column (HiTrap Q HP, GE Healthcare) and gel filtration chromatography (HiLoad_16/60_Superdex200_prep grad, GE Healthcare) using ÄKTApurifier (GE healthcare). The purified protein was dissolved in crystallization buffer (20 mM Tris-HCl, pH 8.0, 200 mM NaCl), identified using SDS–PAGE and crystallized at 10 mg ml^−1^. As the methionine content of PAXX 1–145 is limited, a double-mutated PAXX 1–145 L57M/L105M was designed to prepare the Se-Met-labelled protein. The selenium derivative was expressed using *E. coli* grown in M9 culture with selenio-methionine and purified using the same methods.

### Crystallization and structure determination

Both native and Se-derivative PAXX 1–145 proteins were crystallized by vapour diffusion. The native protein was crystallized in a solution of 1.5 M (NH_4_)_2_SO_4_, 0.1 M Tris-HCl, pH 8.5 and 12% glycerol. Se-derivative crystals were observed in a solution of 2.24 M (NH_4_)_2_SO_4_ and 0.1 M Bis-Tris, pH 5.5. After dehydration and cryo-protection in 50% glycerol, the crystals were flash-cooled using liquid nitrogen. The diffraction data were collected at 100 K at SSRF (Shanghai Synchrotron Radiation Facility) beamline BL17U1 and processed using HKL2000 software. The selenium sites were discovered via SHELX using the single-wavelength anomalous scattering method. The initial phase was generated using AutoSol in PHENIX. AutoBuild was used alternately to improve the electron density map and generate the model. Approximately 90% of the residues in the protein were built automatically and additional missing residues were added manually in COOT and refined with PHENIX. The native structure was determined via Phaser using the MR method. The Se-derivative structure was used as the initial model. The final structure was refined using PHENIX. In the Ramachandran plot generated using PROCHECK, 94.4% and 4.8% of the amino acids in the final atomic model were in the most favourable and additional allowed regions, respectively. All of the structural figures were prepared using COOT and PyMOL.

### Generation of DT40 knockout strains

DT40 knockout constructs for XLF, PAXX, XRCC4 and Lig4 were generated as previously described^36^ using a MultiSite Gateway Three-Fragment Vector Construction Kit. The 5′ and 3′ arms were amplified from genomic DNA using the primers listed in Supplementary Table 2. They were cloned into the pDONR P4-P1R and pDONR P2R-P3 vectors, respectively. The knockout constructs were generated by LR recombination of the pDONR-5′ arm, pDONR-3′ arm, resistance gene cassette-containing pDONR-211 and pDEST R4-R3 destination vector. The knockout constructs were linearized before transfection. The primers used for genomic DNA PCR screening are listed in Supplementary Table 2.

### Generation of HCT116 knockout strains

PAXX-deficient HCT116 cells were generated using CRISPR. Briefly, two guide sequences 5′- CGGGGCGGCTCGGGGCCCGG -3′ and 5′- AGCTGTGGCTCTGACTCTGC -3′, targeting two different sites of the human *PAXX* gene, respectively, were inserted into the pX330 vector^37^. The guide sequence-containing pX330 plasmids were transfected into HCT116 cells. Single colonies were picked after 8–10 days of incubation. The genomic fragments of the *PAXX* gene were amplified by PCR using primers 5′- CCGGCTCCGGAAGTCGTTCTTCC -3′ and 5′- CGGCGTGAAGCAGGTGCTCCAAAG -3′, and 5′- GGTGAGACCCAAGCAGGGAGGAAG -3′ and 5′- GGACCCCAGTGAAGGTATGAGGTG -3′, and digested with ApaI and PstI, respectively. Colonies containing the expected PCR fragments were further sequenced and examined by WB analysis.

### Cell survival assay

The cell survival curves for the DT40 cells were measured using the MTS assay as described previously^38^. Briefly, 1,500–3,000 cells were plated into each well of a 96-well plate with a range of doses of ICRF193 or camptothecin. After a 48-h incubation, the cells were pulsed with CellTiter 96 Aqueous One Solution Reagent (Promega) for 4 h. Cell viability was measured with a luminometer and each dose point was measured in triplicate. For bleomycin and VP16, a density of 300–1,000 cells per well and a 72-h incubation were used.

The IR sensitivity assay for DT40 cells was performed using a colony formation assay. Briefly, 200–20,000 cells were seeded in each well of 6-well plates with 0.7% methylcellulose medium. The plates were exposed to the appropriate dose of X-ray irradiation. After incubating at 39.5 °C for 6 days, the number of colonies was counted. To measure the G1/S phase-specific radiation sensitivity, DT40 cells were cultured in medium containing 0.5 μg ml^−1^ nocodazole for 8 h and then washed three times with PBS containing 5% calf serum. The cells were further cultured in medium containing 0.8 mM mimosine (Sigma) for 8 h and then washed three times as described above. The synchronized cells then were seeded on 0.7% methylcellulose medium for IR.

Colony formation assay of DT40 cells to VP16 was performed as described previously^28^. Briefly, the appropriate number of cells was plated in 0.7% methylcellulose (Sigma)-containing medium with a range of doses of VP16. After incubation at 39.5 °C for 7–14 days, the number of colonies was counted. Cell survival curves for HCT116 cells to IR and VP16 was performed as described^39^. Appropriate cell number was plated in six-well plates, cultured for 24 h and exposed to the appropriate dose of X-ray irradiation. For VP16, the cells were cultured for 24 h and then the indicated dose of VP16 was added to the medium. Colonies were stained with methylene blue and counted after an additional 9–14 days of incubation.

### Electrophoretic mobility shift assay

The DNA substrates were generated by annealing the oligos H40f 5′- GTGACCGTCTCCGGGAGCTGGAAACGCGCGAGACGAAAGG -3′ and H40r 5′- CCTTTCGTCTCGCGCGTTTCCAGCTCCCGGAGACGGTCAC -3′; H60f 5′- GACGCTGCCGAATTCTACCAGTGCCTTGCTGGACATCTTTGCCCACCTGCAGGTTCACCC -3′ and H60r 5′- GGGTGAACCTGCAGGTGGGCAAAGATGTCCAGCAAGGCACTGGTAGAATTCGGCAGCGTC -3′; HL-111 5′- GGGCTACCGTCAAGTAAGATGCAGATACGGAACACAGCTGGCACAGTGGTAGTACTCCACTGTCTGGCTGTACAAAAACCCTCGGGATCT -3′ and HL-112 5′- AGATCCCGAGGGTTTTTGTACAGCCAGACAGTGGAGTACTACCACTGTGCCAGCTGTGTTCCGTATCTGCATCTTACTTGACGGTAGCCC -3′), respectively. Before annealing, the 5′ ends of the oligos H40f, H60f and HL-111 were labelled with ^32^P using T4 polynucleotide kinase. The 30-bp Cy3-labelled double-stranded DNA (dsDNA) was generated as previously described^40^. The 315-bp dsDNA was generated by PCR with primers c9orf142PCRu and c9orf142PCRd, using chicken genomic DNA. The indicated amounts of protein and 5 nM ^32^P- or Cy3-labelled DNA substrate was incubated at 25 °C in 10 μl reaction buffer (25 mM Tris-HCl at pH 7.5, 5 mM MgCl_2_, 100 mM NaCl, 1 mM DTT, 0.05% Triton X-100, 100 μg ml^−1^ BSA and 5% glycerol) for 15 min. The reaction mixture was loaded and resolved in a 5% TBE gel.

### DNA ligation assay

The DNA ligation assay was performed as described previously^16^. Briefly, the reactions were performed in buffer consisting of 25 mM Tris, pH 8.0/100 mM NaCl/0.1 mM EDTA/50 μg ml^−1^ BSA/0.05% Triton X-100/2 mM DTT/5% (wt/vol) PEG8000. The proteins were incubated with DNA substrates for 10 min before the reaction at 25 °C. To initiate ligation, 5 mM MgCl_2_ and 0.1 mM ATP were added to the mixture. The reactions were conducted in 20-μl volumes containing 1 nM DNA (DNA1 and DNA2) and the indicated proteins at 37 °C for 30 min. The reaction was stopped by adding 2 μl of 0.5 M EDTA (pH 8.0). The produced DNA was purified on a QIAquick column (Qiagen) and measured by quantitative PCR.

## Author contributions

M.X., M.Y., W.H., L.W., S.N., Z.Y., W.L., Q.W., C.D. and R.G. conducted the experiments. G.G., J.J., L.L., Y.Land D.X. supervised the project. H.L., F.F., M.X., M.Y., W.H., M.H., G.G., J.J., L.L., Y.L. and D.X. analysed the data. M.X., M.Y., H.L., F.F., Y.L. and D.X. wrote the manuscript, with input from the other authors.

## Additional information

**Accession codes.** The atomic coordinates and structure factors for PAXX N-terminal fragment have been deposited in the Protein Data Bank with accession code 4WJA.

**How to cite this article:** Xing, M. *et al*. Interactome analysis identifies a new paralogue of XRCC4 in non-homologous end-joining DNA repair pathway. *Nat. Commun.* 6:6233 doi: 10.1038/ncomms7233 (2015).

## Supplementary Material

Supplementary Figures and Supplementary Tables Supplementary Figures 1-10 and Supplementary Tables 1-2

Supplementary Data 1 Systematic analysis of non-homologous end joining complexes. The numbers of peptides identified by mass spectrometry are average from two experiments.

## Figures and Tables

**Table 1:** The proteins identified in the purified NHEJ complexes using mass spectrometry.

**Identified proteins**	**Flag-IP**						
	**Mock**	**XRCC4**	**XLF**	**PAXX**	**LIG4**	**Ku70**	**Ku80**
DNA-PKcs	0	0.5	222.5	286.5	270	212	206.5
Ku70	0	1	55	68	71	76.5	62.5
Ku80	0	0	58	62.5	70	73	86
LIG4	0	22.5	44.5	23	94	0	1
XRCC4	0	39	57	14.5	68	4	1
XLF	0	13	27	0	11.5	0	0
PAXX	0	0	5.5	24	8.5	9	8.5
APLF	0	0	2.5	1.5	8	1.5	1
PNKP	0	29	0	1	0	0	0
APTX	0	13	0	0	0	0	0
WRN	0	0	0	37.5	44	15.5	30

APLF, APTX–polynucleotide kinase-phosphatase-like factor; APTX, aprataxin; DNA-PKc, DNA-dependent protein kinase catalytic subunit; IP, immunoprecipitation; Lig4, DNA ligase IV; NHEJ, Non-homologous end joining; PKNP, polynucleotide kinase-phosphatase; XLF, XRCC4-like factor.

The numbers of peptides are averaged over two experiments.

**Table 2:** Data collection and refinement statistics (SAD).

	**Native**	**SeMet**
*Data collection*		
Space group	P6522	P6522
Cell dimensions		
*a*, *b*, *c* (Å)	90.062, 90.062, 153.505	91.825, 91.825, 152.468
*α*, *β*, *γ* (°)	90.000, 90.000, 120.000	90.000, 90.000, 120.000
Wavelength	0.979	0.979
Resolution (Å)	50–2.64 (2.69–2.64)	50–2.80 (2.86–2.80)
*R*_sym_ or *R*_merge_	0.069 (0.634)	0.081 (0.646)
*I*/σ*I*	69.64 (7.52)	56.62 (8.17)
Completeness (%)	99.3 (100)	100 (100)
Redundancy	20.0 (20.9)	22.3 (22.5)
		
*Refinement*		
Resolution (Å)	50–2.64	
No. reflections	10813	
*R*_work_/*R*_free_	23.49/27.06	
No. atoms		
Protein	1947	
Ligand/ion	—	
Water	6	
*B*-factors		
Protein	91.1	
Ligand/ion	—	
Water	67.4	
Root mean squared deviations		
Bond lengths (Å)	0.013	
Bond angles (°)	1.570	

SAD, single wavelength anomalous scattering.
