# Supplementary material for: Interactome analysis identifies a new paralogue of XRCC4 in non-homologous end joining DNA repair pathway
Source: Nat Commun. 2015 Feb 11;6:6233. doi: 10.1038/ncomms7233 (PMC4339890; doi:10.1038/ncomms7233)
Supplement: Supplementary Figures and Supplementary Tables — Supplementary Figures 1-10 and Supplementary Tables 1-2 [file ncomms7233-s1.pdf]

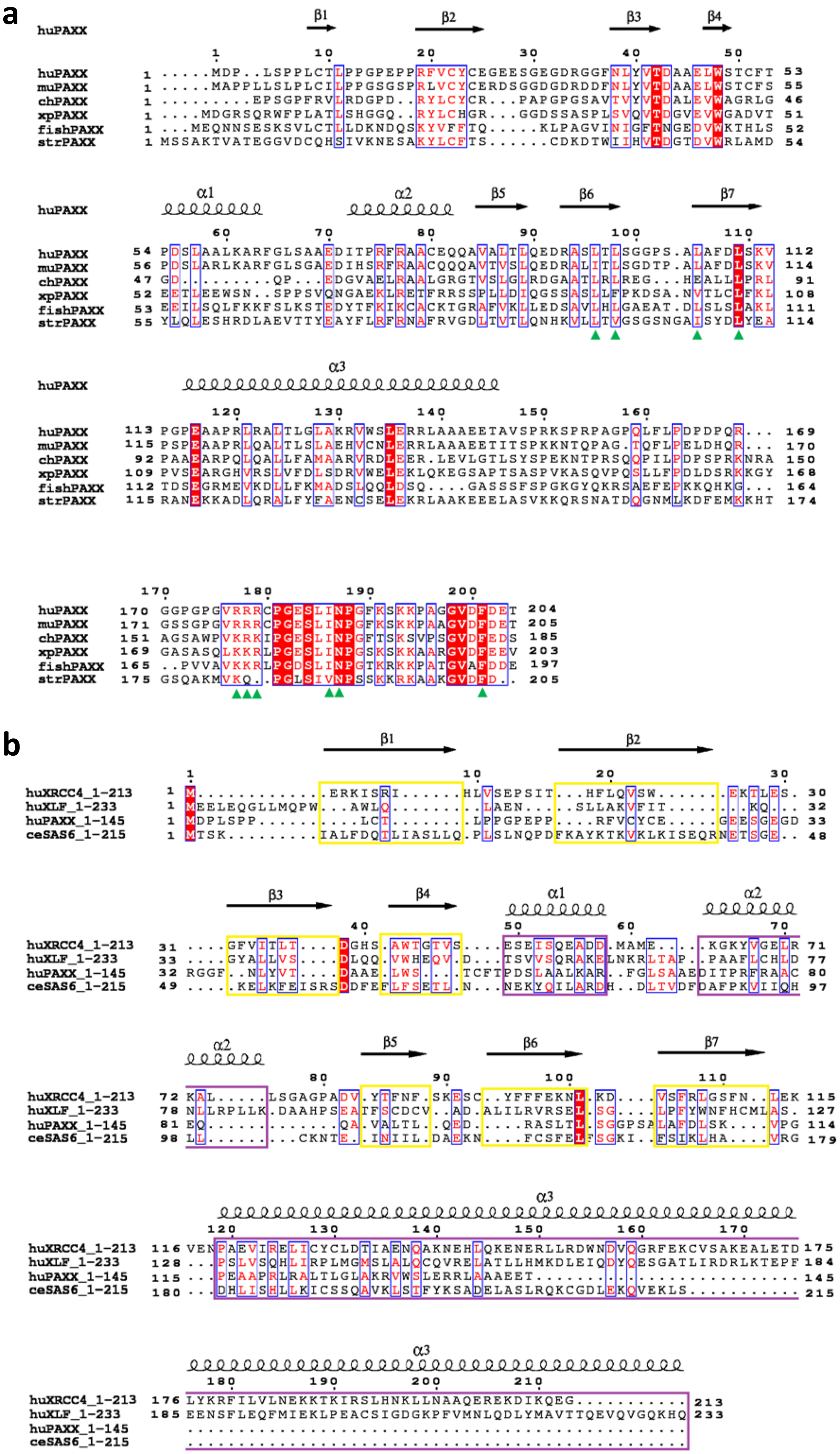
a
huPAXX β1 β2 β3 β4
1 10 20 30 40 50
huPAXX 1 .....MDP..LSPPLCTLPPGPEPPRFVCYCEGEESGEGDRGGFNLYVTDAAELWSTCFT 53
muPAXX 1 .....MAPPLLSLPLCILPPGSGSPRLVCYCERDSGGDGDRDDFNLYVTDAAELWSTCFS 55
chPAXX 1 .........EPSGPFRVLRDGPD..RYLCYCR...PAPGPGSAVTVYVTDALEVWAGRLG 46
xpPAXX 1 ....MDGRSQRWFPLATLSHGGQ..RYLCHGR...GGDSSASPLSVQVTDGVEVWGADVT 51
fishPAXX 1 ...MEQNNSESKSVLCTLLDKNDQSKYVFFTQ.....KLPAGVINIGFTNGEDVWKTHLS 52
strPAXX 1 MSSAKTVATEGGVDCQHSIVKNESAKYLCFTS......CDKDTWIIHVTDGTDVWRLAMD 54
huPAXX α1 α2 β5 β6 β7
60 70 80 90 100 110
huPAXX 54 PDSLAALKARFGLSAAEDITPRFRAACEQQAVALTLQEDRASLTLSGGPS.ALAFDLSKV 112
muPAXX 56 PDSLARLKARFGLSGAEDIHSRFRAACQQQAVTVSLQEDRALITLSGDTP.ALAFDLSKV 114
chPAXX 47 GD.........QP...EDGVAELRAALGRGTVSLGLRDGAATLRLREG...HEALLLPRL 91
xpPAXX 52 EETLEEWSN..SPPSVQNGAEKLRETFRRSSPLLDIQGSSASLLFPKDSA.NVTLCLFKL 108
fishPAXX 53 EEILSQLFKKFSLKSTEDYTFKIKCACKTGRAFVKLLEDSAVLHLGAEAT.DLSLSLAKL 111
strPAXX 55 YLQLESHRDLAEVTTYEAYFLRFRNAFRVGDLTVTLQNHKVLLTVGSGSNGAISYDLYEA 114
huPAXX α3
120 130 140 150 160
huPAXX 113 PGPEAAPRLRALTLGLAKRVWSLERRLAAAEETAVSPRKSPRPAGPQLFLPDPDPQR... 169
muPAXX 115 PSPEAAPRLQALTLSLAEHVCNLERRLAAAEETITSPKKNTQPAG.TQFLPELDHQR... 170
chPAXX 92 PAAEARPQLQALLFAMAARVRDLEER.LEVLGTLSYSPEKNTPRSQQPILPDPSPRKNRA 150
xpPAXX 109 PVSEARGHVRSLVFDLSDRVWELEKLQKEGSAPTSASPVKASQVPQSLLFPDLDSRKKGY 168
fishPAXX 112 TDSEGRMEVKDLLFKMADSLQQLDSQ....GASSSFSPGKGYQKRSAEFEPKKQHKG... 164
strPAXX 115 RANEKKADLQRALFYFAENCSELEKRLAAKEEELASVKKQRSNATDQGNMLKDFEMKKHT 174
170 180 190 200
huPAXX 170 GGPGPGVRRRCPGESLINPGFKSKKPAGGVDFDET 204
muPAXX 171 GSSGPGVRRRCPGESLINPGFKSKKPAAGVDFDET 205
chPAXX 151 AGSAWPVKRKIPGESLINPGFTSKSVPSGVDFEDS 185
xpPAXX 169 GASASQLKKRLPGESLINPGSKSKKAARGVDFEEV 203
fishPAXX 165 ..PVVAVKKRLPGDSLINPGTKRKKPATGVAFDDE 197
strPAXX 175 GSQAKMVKQ..PGLSIVNPSSKKRKAAKGVDFD.. 205
b
β1 β2
1 10 20 30
huXRCC4_1-213 1 M............ERKISRI......HLVSEPSIT...HFLQVSW.......EKTLES. 30
huXLF_1-233 1 MEELEQGLLMQPW...AWLQ........LAEN.....SLLAKVFIT.........KQ.. 32
huPAXX_1-145 1 MDPLSPP.........LCT........LPPGPEPP....RFVCYCE.....GEESGEGD 33
ceSAS6_1-215 1 MTSK..........IALFDQTLIASLLQ.PLSLNQPDFKAYKTKVKLKISEQRNETSGE.. 48
β3 β4 α1 α2
40 50 60 70
huXRCC4_1-213 31 ....GFVITLT....DGHS.AWTGTVS....ESEISQEADD.MAME.....KGKYVGELR 71
huXLF_1-233 33 ....GYALLVS....DLQQ.VWHEQV.D...TSVVSQRAKELNKRLTAP..PAAFLCHLD 77
huPAXX_1-145 32 RGGF.NLYVT....DAAE.LWS....TCFTPDSLAALKAR.FGLSAAEDITPRFRAAC 80
ceSAS6_1-215 49 .....KELKFEISRSDDFEFLFSETL.N...NEKYQILARDH..DLTVDFDAFPKVIIQH 97
α2 β5 β6 β7
80 90 100 110
huXRCC4_1-213 72 KAL.....LSGAGPADV.YTFNF.SKESC..YFFFEKNL.KD....VSFRLGSFN..LEK 115
huXLF_1-233 78 NLLRPLLK.DAAHPSEATFSCDCV..AD.ALILRVRSEL.SG....LPFYWNFHCMLAS. 127
huPAXX_1-145 81 EQ..............QA.VALTL..QED.....RASLTL.SGGPSALAFDLSK....VPG 114
ceSAS6_1-215 98 LL.......CKNTE..INIIL.DAEKN....FCSFELFSGKI..FSIKLHA....VRG 179
α3
120 130 140 150 160 170
huXRCC4_1-213 116 VENPAEVIRELICYCLDTIAENQAKNEHLQKENERLLRDWNDVQGRFEKCVSAKEALETD 175
huXLF_1-233 128 ...PSLVSQHLIRPLMGMSLALQCQVRELATLLHMKDLEIQDYQESGATLIRDRLKTEPF 184
huPAXX_1-145 115 ...PEAAPRLRALTLGLAKRVWSLERRLAAAEET.......................... 145
ceSAS6_1-215 180 ...DHLISHLLKICSSQAVKLSTFYKSADELASLRQKCGDLEKQVEKLS........... 215
α3
180 190 200 210
huXRCC4_1-213 176 LYKRFILVLNEKKTKIRSLHNKLLNAAQEREKDIKQEG............ 213
huXLF_1-233 185 EENSFLEQFMIEKLPEACSIGDGKPFVMNLQDLYMAVTTQEVQVGQKHQ 233
huPAXX_1-145 ....
ceSAS6_1-215 ....

Supplementary Figure 1. Sequence Conservation of the N-Terminal Region of PAXX.

(**a**) Sequence alignment of PAXX homologues. The PAXX orthologues are identified using the BLASTP algorithm, which searches the NR database maintained by NCBI. They are from human (huPAXX, *Homo sapiens* NP_899064.1), mouse (muPAXX, *Mus musculus* NP_705785.1), chicken (chPAXX, *Gallus gallus* XP_001233897), *Xenopus (Silurana) tropicalis* (xpPAXX, XP_004917607.1), zebrafish (fishPAXX, *Danio rerio* NP_001124069.1) and *Strongylocentrotus purpuratus* (strPAXX, XP_799995.3). The identical amino acids are shaded in red; the similar amino acids are highlighted with red letters. Secondary structure elements are indicated above. The residues mutated in Nmut (Fig. 4A), 177AAA, 186AA and 201A (Fig. 3A) are marked with green stars. (**b**) Structure-based sequence alignment of PAXX (1-145), XLF(1–233) and XRCC4(1–211). Secondary structure elements are indicated above. Purple and yellow boxes indicate amino acids contributing to α-helixes and β-sheets, respectively.

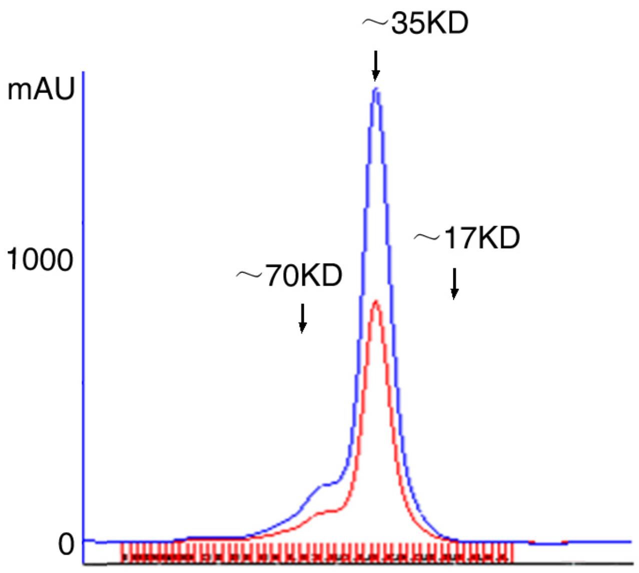

Supplementary Figure 2. The aggregation states of PAXX 1-145 in solution. A HiLoad_16/60_ Superdex200_prep grad gel filtration column was used. Both wavelengths at 280 nm (shown in blue) and 254 nm (shown in red) were detected. Supposed positions for monomer (17KD), dimer (35KD) and tetramer (70KD) are shown.

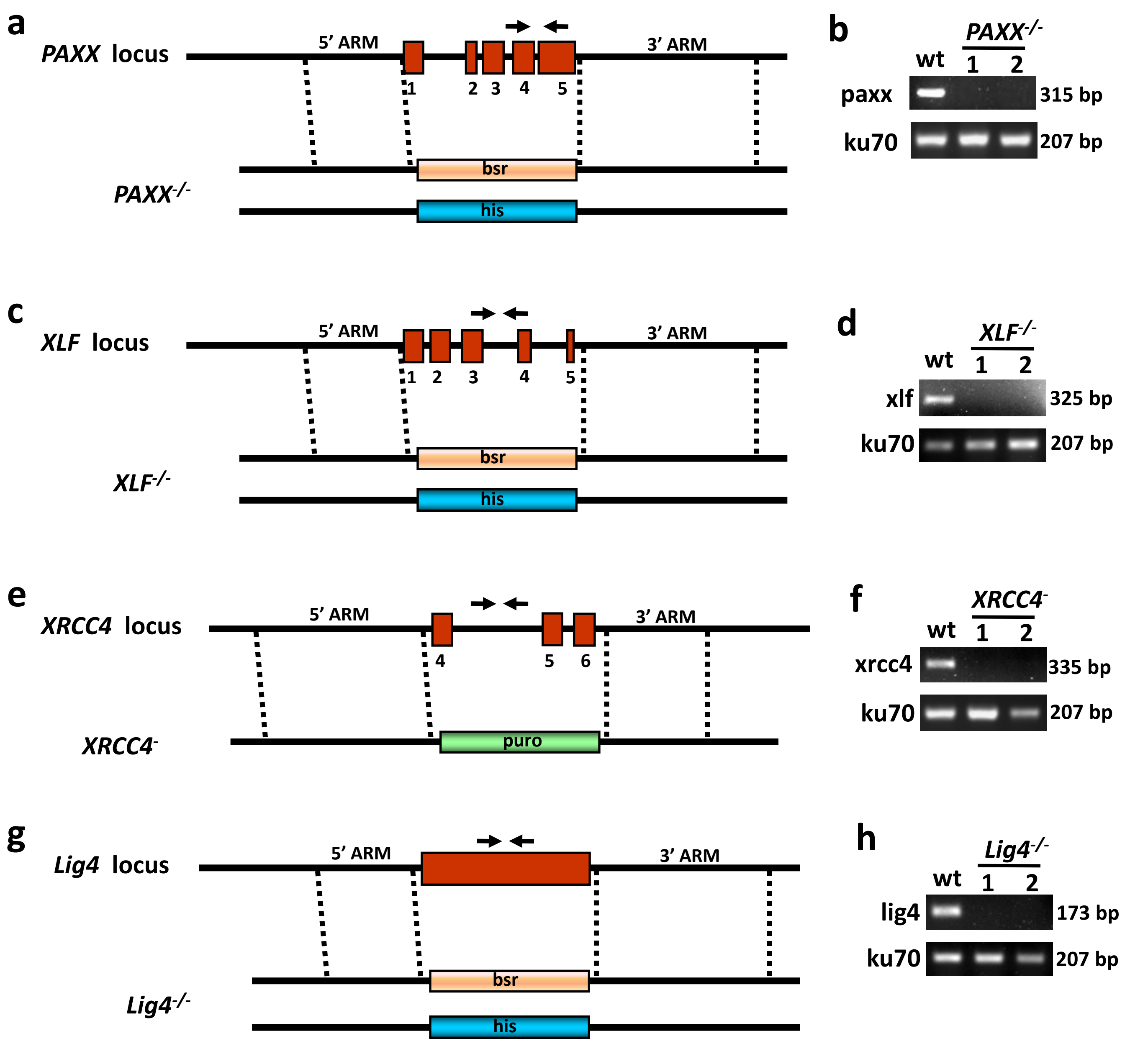

Supplementary Figure 3. Generation of PAXX, XLF, XRCC4 and Lig4 knockout DT40 cells. (**a**) (**c**) (**e**) (**g**) Schematic representation of the chicken PAXX (**a**), XLF (**c**), XRCC4 (**e**) and Lig4 (**g**) wild-type and targeted genomic DNA. The regions containing exons (marked by red) of these genes are replaced by blasticidine, histidinol or puromycin resistant gene. The two regions between two pairs of dotted lines were used as arms of the knock-out constructs. Arrows indicated targeted locations of primers for genomic PCR. (**b**) (**d**) (**f**) (**h**) Genome-PCR analysis to show that PAXX (**b**), XLF (**d**), XRCC4 (**f**) and Lig4 (**h**) genes are undetectable in the respective knockout DT40 cells. Ku70 was included as a positive control.

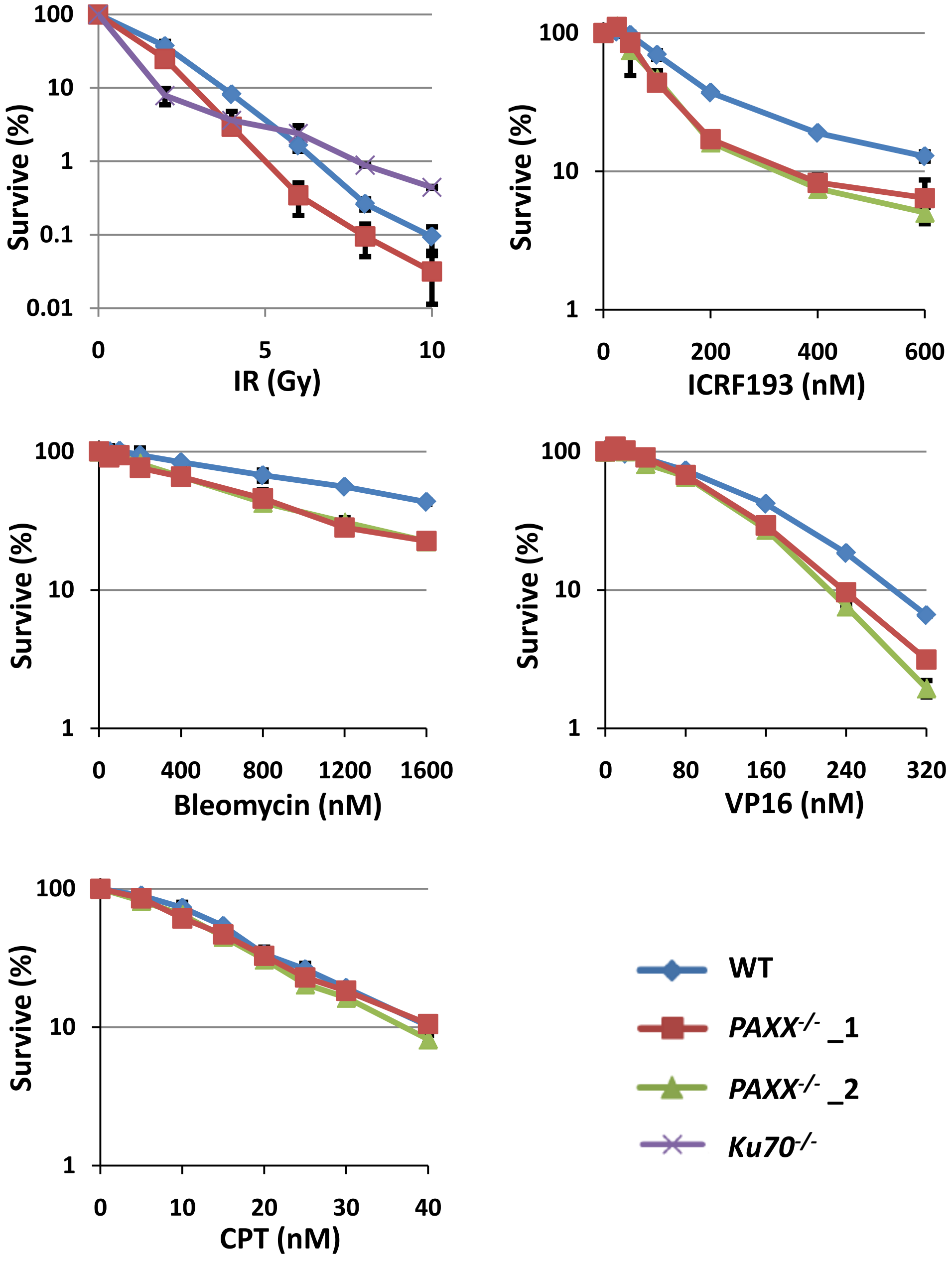

Supplementary Figure 4. PAXX is required for DSB repair. Sensitivity curves of PAXX null DT40 cells to Bleomycin, VP16, ICRF193 and CPT. Cell survival curves were measured by using MTS assay. Cells are asynchronized. Mean and s.d. from three independent experiments are shown. Two different clones are tested for the PAXX mutants.

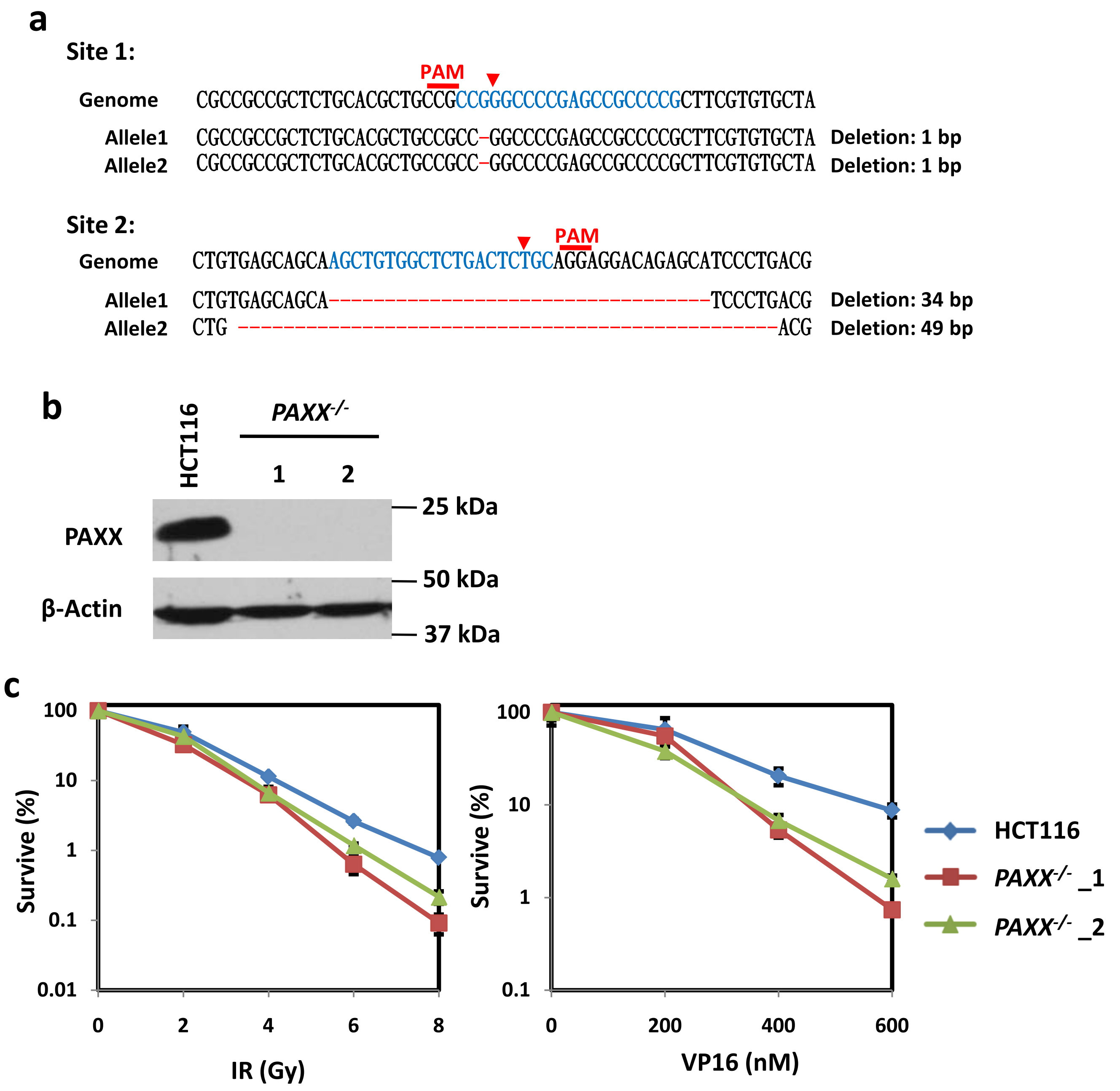

Supplementary Figure 5. PAXX is required for DSB repair in HCT116 cells. (**a**) Schematic representation of the generation of *PAXX*$^{-/-}$ HCT116 cells in two different sites using CRISPR. The guide sequences are highlight by blue. PAM sequences are indicated by red lines. Red arrow indicates putative cleavage site. Red dashes, deleted bases. (**b**) Immunoblotting shows that PAXX protein is absent in two *PAXX*$^{-/-}$ HCT116 cell clones. β-Actin is included as a control. (**c**) Sensitivity curves of the *PAXX*$^{-/-}$ HCT116 cells to IR and VP16. Mean and s.d. from three independent experiments are shown.

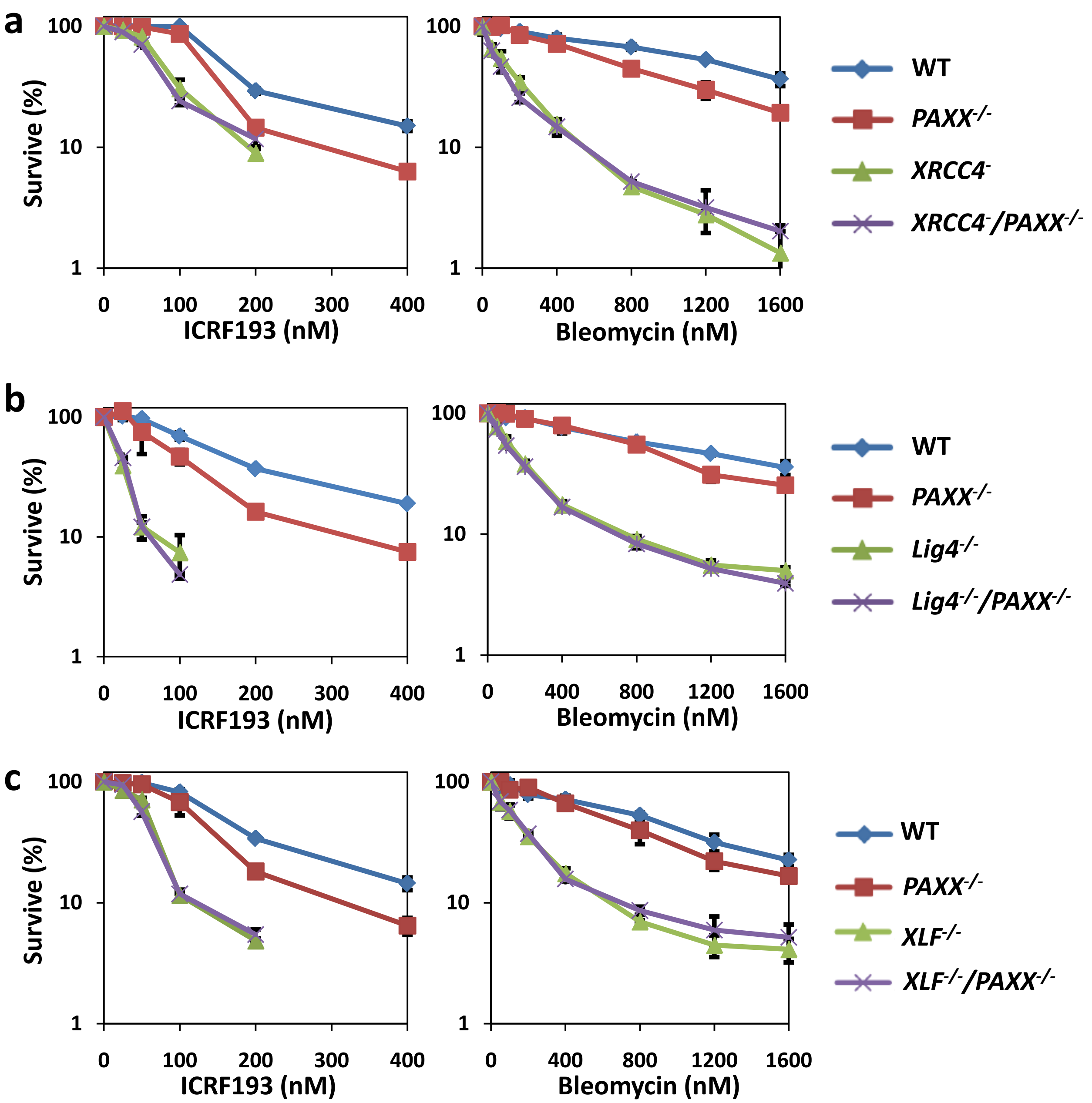

Supplementary Figure 6. PAXX and XLF show both epistasis and non-epistasis in DSB repair. (**a, b, c**) Sensitivity assays analysed genetic interactions of PAXX with XRCC4 (**a**), Lig4 (**b**) and XLF (**c**). Cell survival curves were measured by using MTS assay. Cells are asynchronized. Mean and s.d. from three independent experiments are shown.

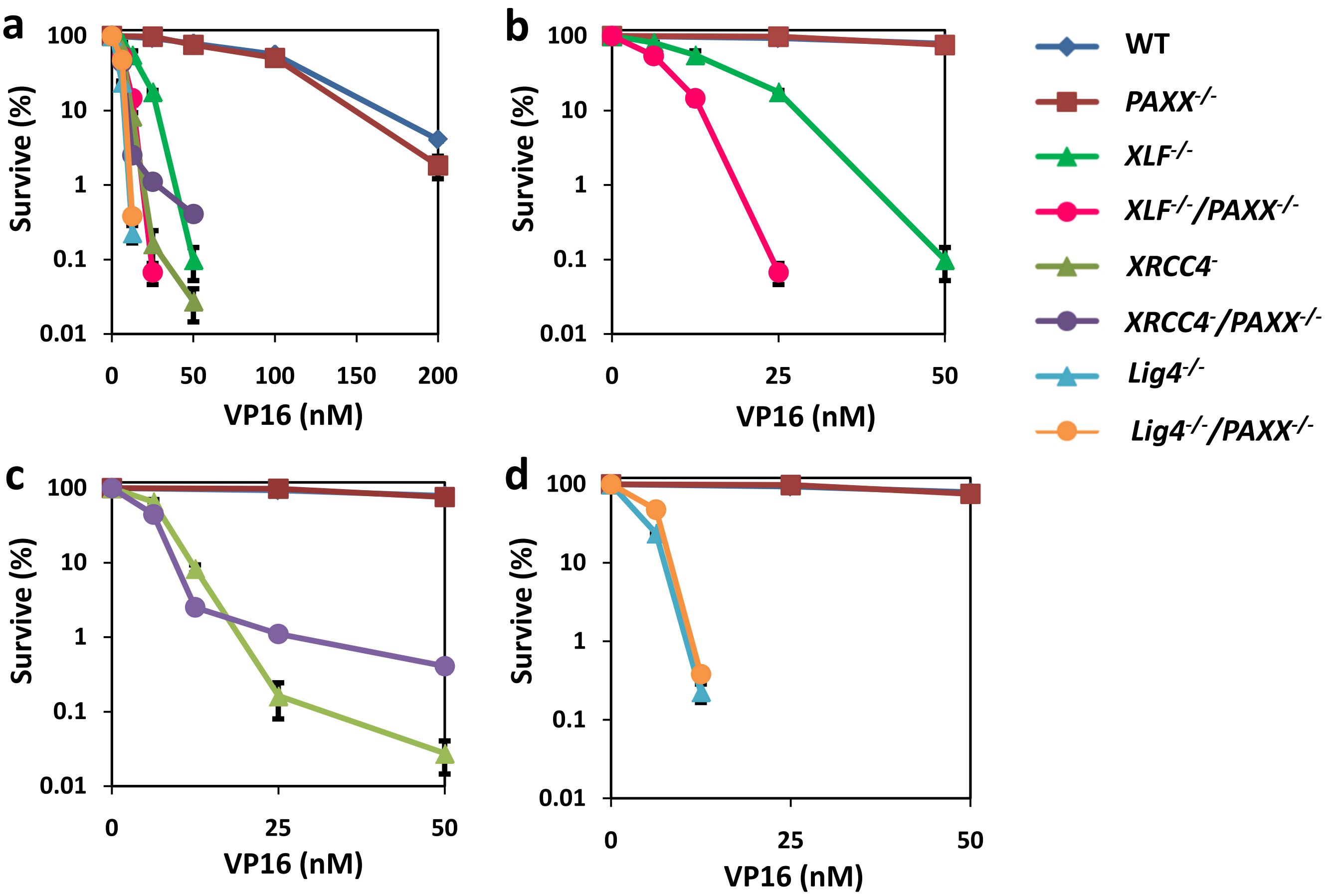

Supplementary Figure 7. Colony formation assay of DT40 cells to VP16. (**a**) Genetic interaction analysis of PAXX with XRCC4, Lig4 and XLF using colony formation assay. (**b, c, d**) To distinguish the curves, panel (**a**) was separated to three other panels (**b**), (**c**) and (**d**). Cells are asynchronized. Mean and s.d. from two independent experiments are shown.

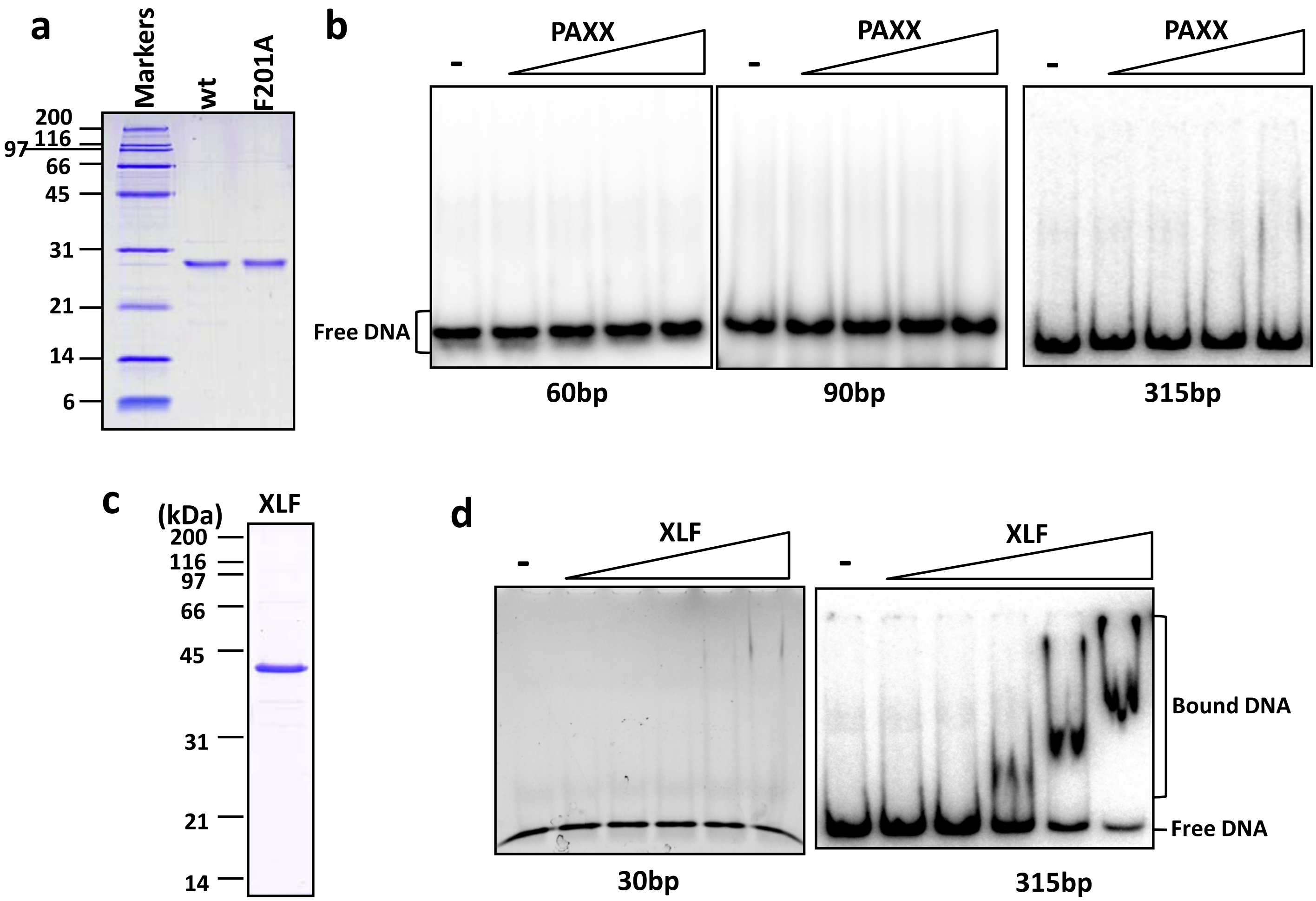

Supplementary Figure 8. PAXX has no DNA binding activity. (**a**) (**c**) Coomassie blue-stained SDS gels show purified PAXX (**a**) and XLF protein (**c**). 1 μg of each respective protein was loaded. (**b**) (**d**) Gel-shift assays show the DNA binding activity of PAXX (**b**) and XLF (**d**) with various dsDNAs illustrated at the bottom. Reactions contained 5 nM of the indicated ^{32}P or Cy3-labelled substrates and 10 nM, 30 nM, 100 nM and 300 nM purified PAXX (**b**) or 25 nM, 50 nM, 100 nM, 200 nM and 400 nM purified XLF (**d**). The protein–DNA complexes were analysed by 4% or 5% polyacrylamide gels.

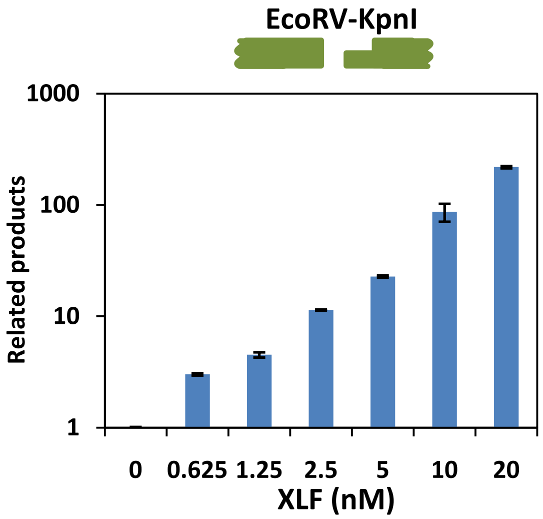

Supplementary Figure 9. XLF stimulates non-cohesive end joining. The linear DNA substrates contained blunt-3’ overhanging (EcoRV-KpnI) ends. Reactions contained 5 nM Ku70/80, 2.5 nM XRCC4-Lig4 and the indicated concentration of XLF. Data from three independent experiments are represented as the mean ± SEM.

Supplementary Figure 10. Uncropped immunoblots

Figure 1b Raw data:

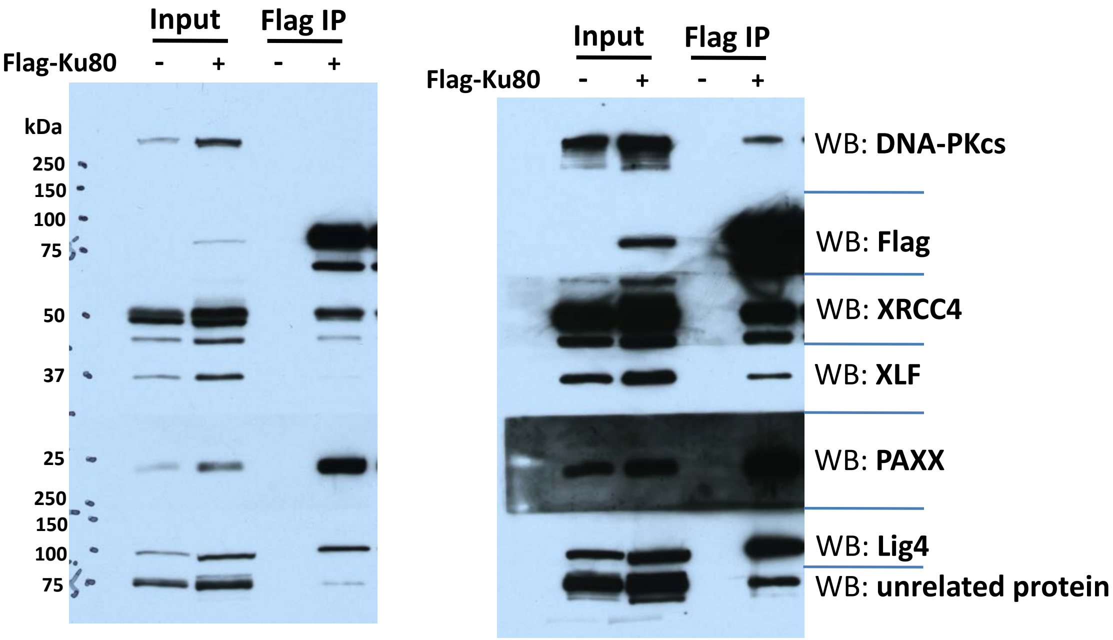

Light exposure

long exposure

Figure 1c Raw data:

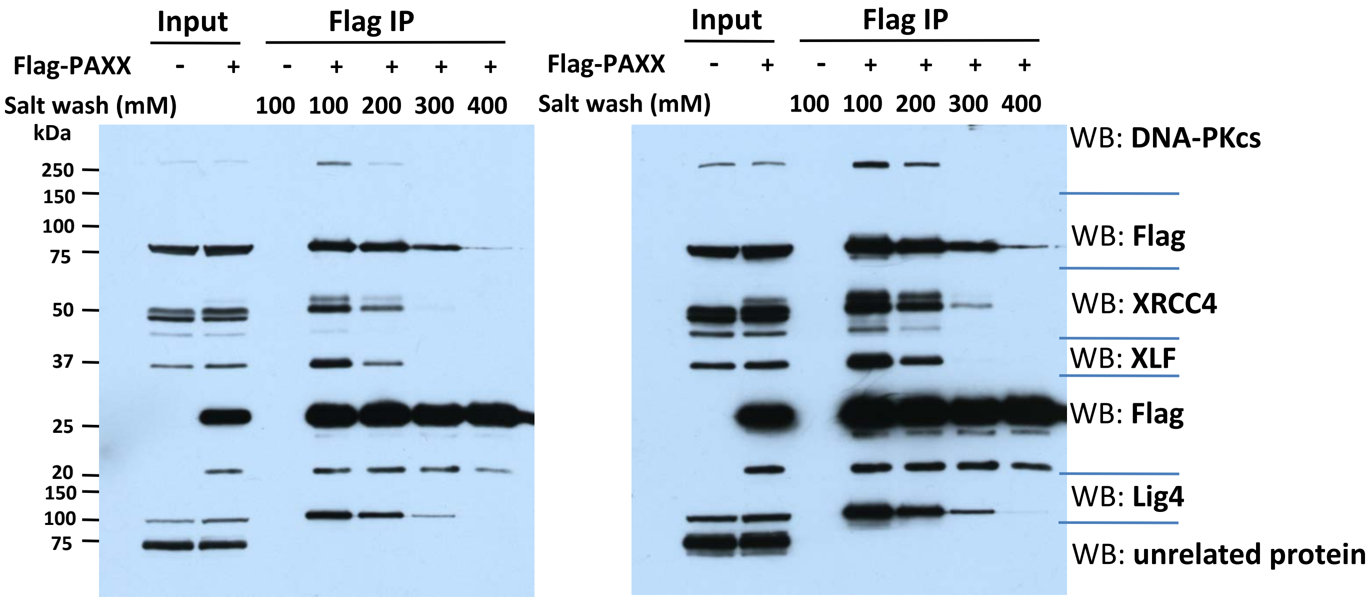

Light exposure

long exposure

Figure 1e Raw data:

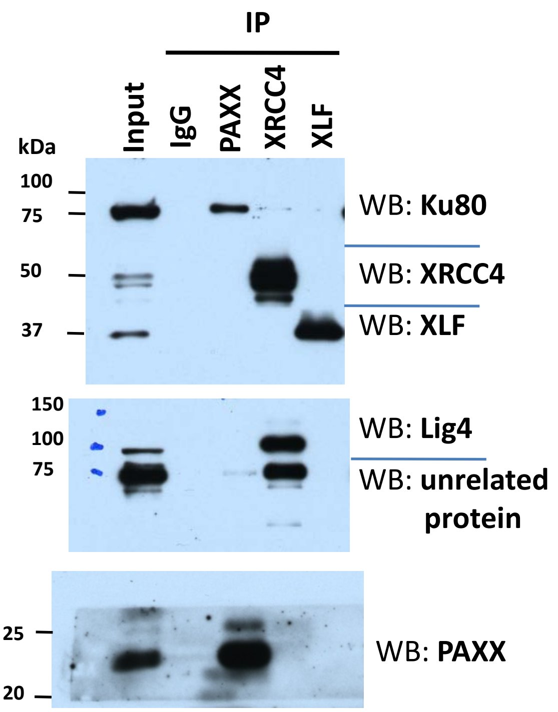

Figure 3b Raw data:

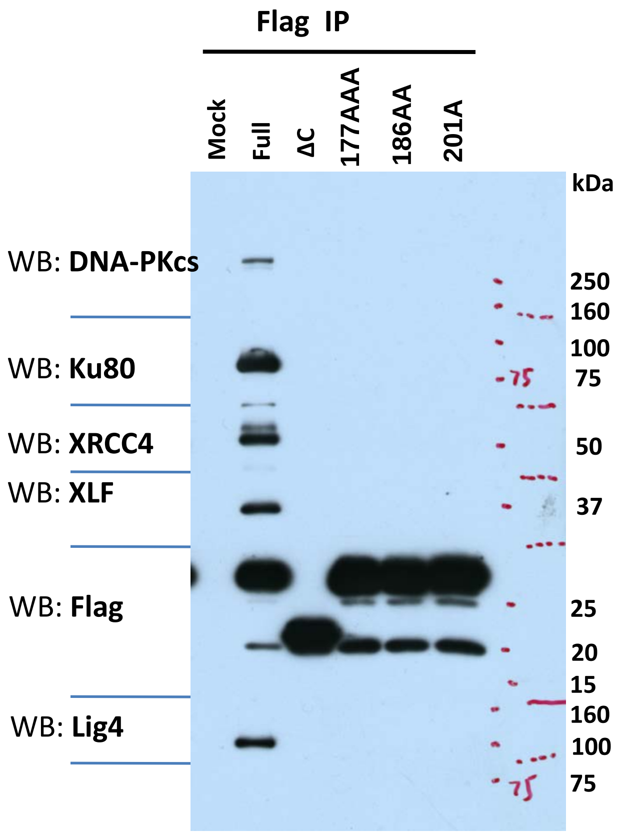

Figure 5a Raw data: (XLF2=PAXX)

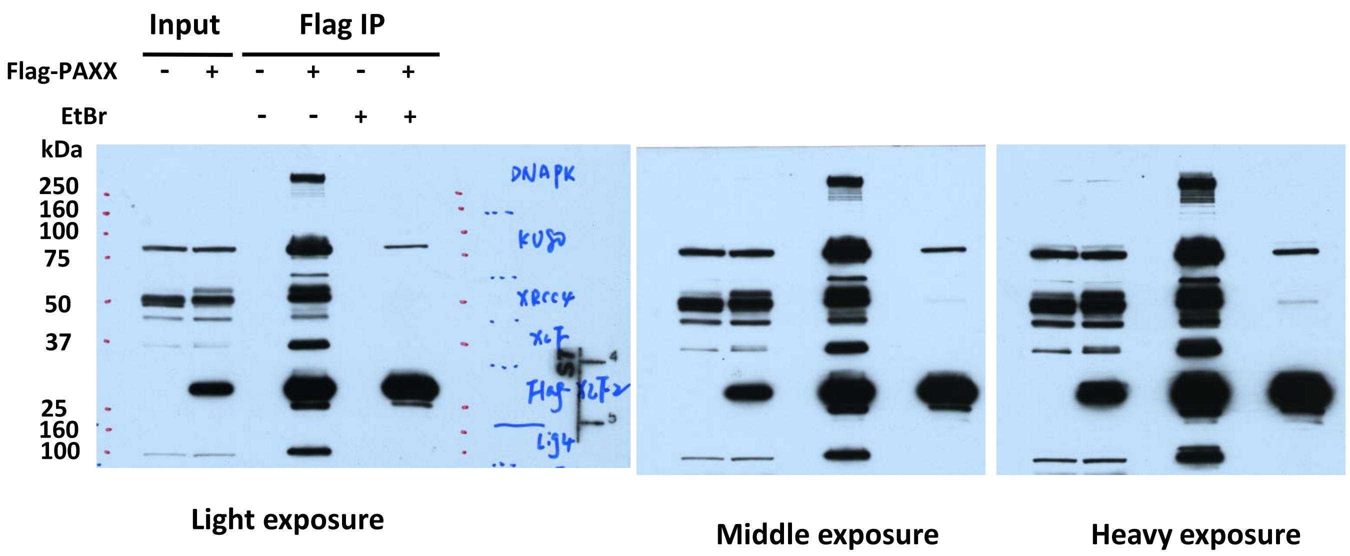

Supplementary Figure 5b Raw data:

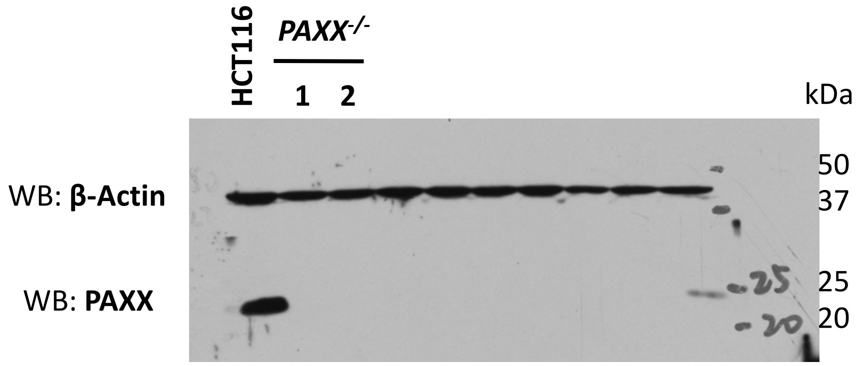

Supplementary Table 1.
Doubling times of the various knockout DT40 cells

	Doubling time (hours)
WT	8.35±0.21
PAXX$^{-/-}$	8.89±0.36
XLF$^{-/-}$	9.32±0.70
PAXX$^{-/-}$/*XLF*$^{-/-}$	10.20±0.51
XRCC4^{-}	9.47±0.42
PAXX$^{-/-}$/*XRCC4*$^{-}$	11.32±0.92
Lig4$^{-/-}$	11.03±0.41
PAXX$^{-/-}$/*Lig4*$^{-/-}$	9.63±0.45

Supplementary Table 2. Primers for DT40 cell knockout

Primer Name	Primer sequence	Description
c9orf142ARM1u	GAGA ACA ACT TTG TAT AGA AAA GTT G acgtgtgcaggtctggagacatttcac	for PAXX 5` arm
c9orf142ARM1d	GAGA AC TGC TTT TTT GTA CAA ACT TG gcactcactagacggtgacggccgag	for PAXX 5` arm
c9orf142ARM2u	GAGA ACA GCT TTC TTG TAC AAA GTG G caagcagcaccaggtggacccgatg	for PAXX 3` arm
c9orf142ARM2d	GAGA AC AAC TTT GTA TAA TAA AGT TG atatc TTTCACATGCTGGCACCAGCTGTTTCTC	for PAXX 3` arm
c9orf142PCRu	cggctcagtgctcctgaggtgtg	for genomic DNA PCR of PAXX
c9orf142PCRd	ggttaatgagggactctcctgggatc	for genomic DNA PCR of PAXX
XRCC4ARM1u	GAGA ACA ACT TTG TAT AGA AAA GTT G ttcccaaagtagcttctaccagcag	for XRCC4 5` arm
XRCC4ARM1d	GAGA AC TGC TTT TTT GTA CAA ACT TG gtattggcatgtgatcttttgttcag	for XRCC4 5` arm
XRCC4ARM2u	GAGA ACA GCT TTC TTG TAC AAA GTG G cttcattttactaaggaaggtatagc	for XRCC4 3` arm
XRCC4ARM2d	GAGA AC AAC TTT GTA TAA TAA AGT TG ctcgag caagaccactcactgattgacttaag	for XRCC4 3` arm
XRCC4gPCRu	gttgatactgcctggacattaagaagc	for genomic DNA PCR of XRCC4
XRCC4gPCRd	gcattactgtgaaactatcacacttg	for genomic DNA PCR of XRCC4
XLFarm1U	GAGA ACA ACT TTG TAT AGA AAA GTT G cgtgttgcaaagcagcgatgtgtg	for XLF 5` arm
XLFarm1D	GAGA AC TGC TTT TTT GTA CAA ACT TG cagaaggtttgcgtggctaaaatatgg	for XLF 5` arm

XLFarm2U	GAGA ACA GCT TTC TTG TAC AAA GTG G gatgaatgaggagatgccatctgctc	for XLF 3` arm
XLFarm2D	GAGA AC AAC TTT GTA TAA TAA AGT TG ctcgag catggcagagtacagcacagtgtgttac	for XLF 3` arm
XLFgPCRu	ctcctggtttttcacttaatgtagcc	for genomic DNA PCR of XLF
XLFgPCRd	ctgcaaagccaggctcatccgtaccag	for genomic DNA PCR of XLF
Lig4Arm1_u	GAGA ACA ACT TTG TAT AGA AAA GTT G atatc gttgcagccctatctacctgtcttc	for Lig4 5` arm
Lig4Arm1_d	GAGA AC TGC TTT TTT GTA CAA ACT TG CAGGTGCGGAAGCCATCCAGGTTG	for Lig4 5` arm
Lig4Arm2_u	GAGA ACA GCT TTC TTG TAC AAA GTG G ggaaggagtcccaaagaatgaaactcag	for Lig4 3` arm
Lig4Arm2_d	GAGA AC AAC TTT GTA TAA TAA AGT TG CTGCAAGAGTGCCTGATGCAGCAAG	for Lig4 3` arm
Lig4gPCRu	agccagaatatgtcaacggg	for genomic DNA PCR of Lig4
Lig4gPCRd	AGAGCCAACACGACAAATAGAG	for genomic DNA PCR of Lig4
